# Whole-genome mapping reveals QTLs linked to key agronomic traits in bi-parental populations of field cress (*Lepidium campestre*)

**DOI:** 10.1186/s12870-025-06197-3

**Published:** 2025-02-24

**Authors:** Kibrom B. Abreha, Cecilia Hammenhag, Felix Seifert, Mulatu Geleta

**Affiliations:** 1 Department of Plant Breeding, Swedish University of Agricultural Sciences, Alnarp, 234 22 Sweden; 2 cropSeq bioinformatics, 22043 Hamburg, Germany

**Keywords:** *De novo* domestication, Domestication-syndrome traits, Genotyping-by-sequencing, Linkage group, SeqSNP, Single nucleotide polymorphism

## Abstract

**Background:**

Field cress, *Lepidium campestre*, is an oil and catch crop undergoing domestication for the Nordic region. In this study, the genetic bases of domestication-related traits of field cress are identified using three bi-parental F_2_ mapping populations (MPs). The MPs were phenotyped for plant height (PH), inflorescence length (IL), pod density (PD), seed yield per plant (SYPP), seed dormancy (SD), and pod shattering measured with two different parameters (PSH1 and PSH2).

**Results:**

The MPs were genotyped, with a targeted Genotyping-by-sequencing (GBS) method, SeqSNP, using 9,378 Single Nucleotide Polymorphisms (SNP) spanning across eight linkage groups (LGs) of field cress. There was wide phenotypic variation among the individuals for the agronomic traits measured in all MPs. A linkage map was constructed for each MP by mapping high-quality SNPs spanning 607 cM, 893 cM, and 732 cM to the eight field cress LGs, in each of the respective MPs. Quantitative trait loci (QTLs) mapping identified nine QTLs linked to PSH2, three for PH, two for SYPP, and one each for SD, IL, and PD distributed across all LGs. Taking advantage of field cress genome synteny with *Arabidopsis thaliana* chromosomes, annotation of the genes found within a major QTL for PSH2 found in LG5 (for MP3) revealed putative roles related to flowering, seed, and siliques development, cellulose and lignin biosynthesis, and water loss prevention.

**Conclusion:**

This study identified QTLs for multiple domestication-related traits and provides genomic resources useful for applying novel breeding tools to accelerate field cress domestication and improvement.

**Supplementary Information:**

The online version contains supplementary material available at 10.1186/s12870-025-06197-3.

## Background

Utilizing the genetic diversity within the few cultivated plant species [1] and their wild relatives is not enough to mitigate the impact of changing climate, continue enhancing productivity, expand into new production areas, and meet the demands for new quality traits. Domesticating new plant species with desirable traits is needed to add novel crops to cultivation, to promote resilience in agricultural systems, and provide new products for human and animal consumption, and industrial inputs [2].

Field cress (*Lepidium campestre* (L.) R. Br) of the Brassicaceae family has been targeted for domestication as a novel oil crop for industrial applications [3] because of its excellent winter hardiness, high seed yield, self-fertilization, and small genome [4–6]. *L. campestre* can be grown as a catch crop to reduce soil erosion and nutrient leaching from agricultural soils and improve crop yield [7], produce oil for industrial applications [4], and serve as a protein source for feed [6, 8]. *L. campestre* is self-pollinated [3, 9], and it can be cross hybridized with closely related wild *Lepidium* species: *L. heterophyllum* Benth. and *Lepidium hirtum* (L.) Sm [10]. Moreover, it has a small genome of 168.1Mbp (unpublished) organized into eight chromosomes (2n = 16) [11]. It is closely related and shares conserved synteny with the model plant species *Arabidopsis thaliana* [9, 10]. Its convenience for breeding and potential as a novel oil crop coupled with the presence of wild relatives that could serve as a source of desirable traits make *L. campestre* an ideal candidate for domestication. However, domestication is time-consuming, labour-intensive, and costly [9], as it requires the improvement of multiple characteristics referred to as “domestication syndrome” traits, such as crop vigor and productivity [1]. Hence, developing efficient and cost-effective breeding techniques to accelerate the domestication of novel crops, such as *L. campestre* is highly desirable [3, 9].

Gustafsson et al. [9], reported polymorphisms in 30 homologs of *A. thaliana* genes that regulate traits targeted in *L. campestre* domestication. Adventitious shoot regeneration [12] and transformation [13] methods for this prototype crop have been developed. CRISPR/Cas9-mediated genome editing has also been successfully applied to improve the fatty acid composition in its seed oil [14, 15]. Using direct gene silencing, it was also possible to increase oleic acid content and reduce pod shattering [16, 17]. The results suggest that *L. campestre* is well-suited to develop, adopt, and apply state-of-the-art new breeding tools to understand its genetic variation, accurately identify genotypes with desirable traits, accelerate its domestication, and subsequently improve key agronomic characteristics. New breeding tools are also crucial for avoiding linkage drag and for maintaining existing fitness advantage and genetic diversity, which could otherwise be lost during domestication [1, 18, 19].

Most domestication-targeted traits are complex traits regulated by multiple genes spanning across a genome [20–22]. Genetic linkage-based quantitative trait loci (QTLs) mapping is a fundamental step in identifying a set of genetic marker–trait associations, and genes governing key traits for domestication [21, 23, 24]. Recently, a high-density genetic linkage map comprising eight linkage groups has been constructed for *L. campestre* [10]. QTLs for some domestication-related traits, such as plant height, number of stems per plant, and perenniality have also been detected [24]. Detecting QTLs is invaluable for dissecting the genetic basis of a trait under investigation and provides targets for applying new breeding techniques. Genetic markers associated with traits of interest can be validated and applied in marker-assisted selection (MAS)-based breeding, as shown in several crops including members of the *Brassicaceae* family, to improve morphological traits [25], disease resistance [26], and oil content [27]. Although the MAS approach is instrumental for efficient and accelerated domestication and subsequent improvement, it has not been applied to *L. campestre* due to limited knowledge of the QTLs and genetic markers governing domestication-targeted traits. Advancements in high-throughput next-generation sequencing (NGS) technologies and bioinformatics make it possible to conduct genotyping-by-sequencing (GBS) on diverse plant species and detect sequence variants across their genomes. GBS has been widely used for developing single nucleotide polymorphism (SNP) markers, which are versatile due to their abundance across genomes and reliability [28, 29]. SNP markers have been employed to understand genetic diversity and population structure, identify genomic regions linked to desirable traits, and used as genetic markers in Markers-assisted breeding for crop improvement [30–34]. Hence, identifying SNP markers would be critical for advancing genomic research and accelerating the domestication process of *L. capmestre*.

This study is aimed at developing bi-parental mapping populations and genotyping them using genotyping by sequencing methods and phenotyping them for key agro-morphological and physiological traits, to determine genomic regions containing genes regulating domestication-targeted traits through QTL mapping using polymorphic SNP markers. The study is also aimed at determining the contribution of SNP variants flanking major QTL regions for the target traits. This was to gain insight into the genetic basis governing key target traits and develop genomic resources required for the fast-track domestication of *L. campestre* and its subsequent improvement.

## Materials and methods

### The plant material and phenotyping

For this study, three F_2_ mapping populations (MPs) were developed: MP1, MP2 and MP3. MP1’s parents are *LP-92-1* and *Trell-1*. *LP-92-1* is characterized by high seed dormancy and pod-shatter resistance whereas *Trell-1* exhibits low seed dormancy and high susceptibility to pod shattering. *Trell-1* is taller and yields lower than *LP-92-1* when grown under the same environmental conditions. The parents of MP2 are *Merb-3* and *LP-92-6*. *Merb-3* is highly susceptible to pod shattering, whereas *LP-92-6* exhibits high resistance. When grown under the same environmental conditions, *Merb-3* produces a longer inflorescence and is taller and more productive than *LP-92-6*. *HH-1-20* and *Hy-14 A* are the parents of MP3. *Hy-14 A* is highly susceptible to pod shattering, while *HH-1-20* is resistant. *HH-1-20* is shorter in PH and IL and has lower SYPP and PD than *Hy-14 A* when grown under the same environmental conditions.

**Fig. 1:**
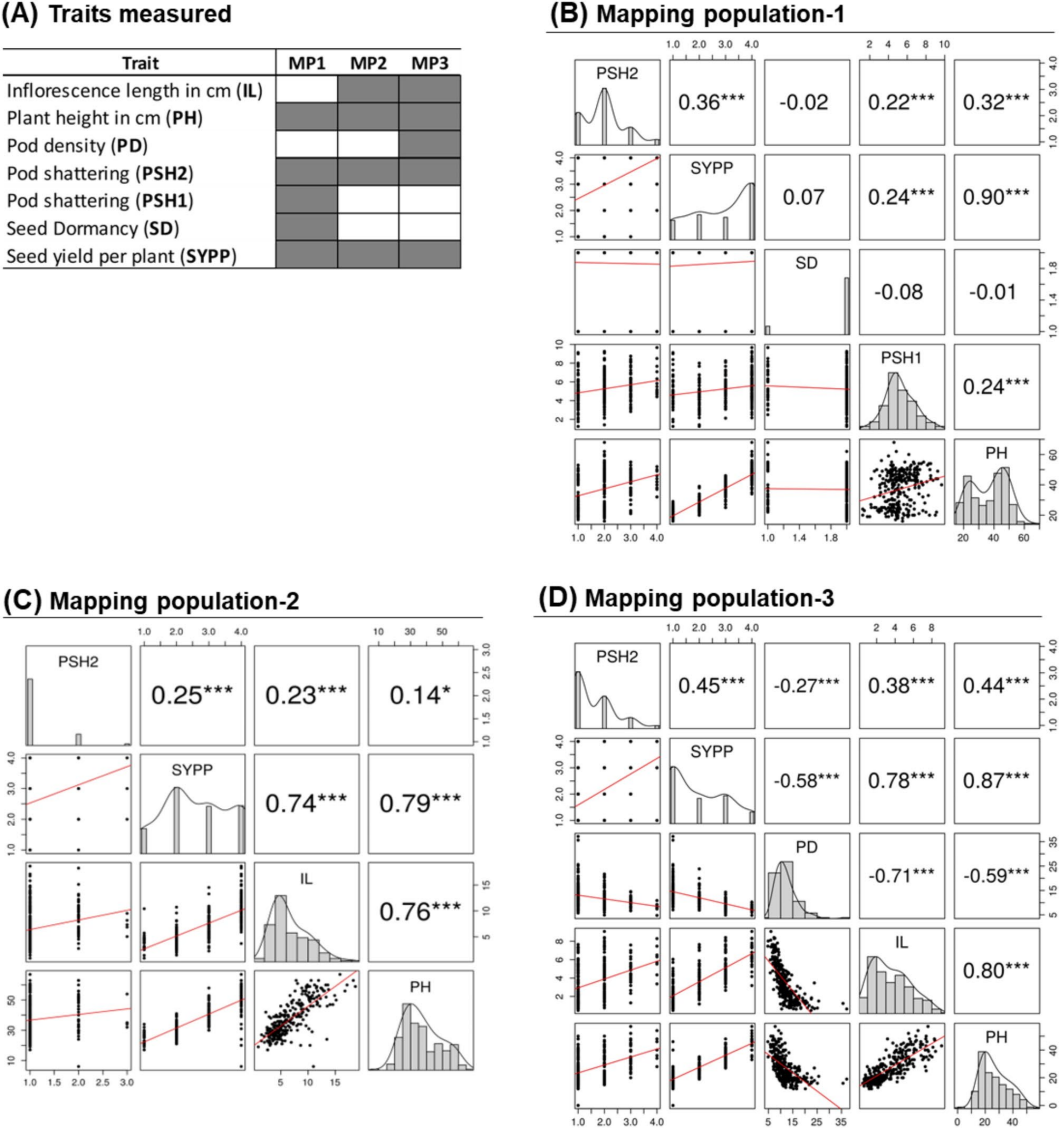
Distribution and correlation analyses of traits targeted in this study in three F_2_ mapping populations (MPs) of *L. campestre*. Diagrammatic representations of traits studied (grey) and not studied (white) in each mapping population (**A**). Density distribution of the traits measured in each MP, scatterplots for each pair of traits, linear correlation (red lines) and Pearson’s correlation coefficients among the traits in MP1 (**B**), MP2 (**C**), and MP3 (**D**). Significance levels of Pearson’s correlation coefficients: * = *p* < 0.05, ** = *p* < 0.01, and *** = *p* < 0.001. PSH1 and PSH2 = pod shattering-1 and 2, SD = seed dormancy, IL = inflorescence length, PD = pod density, PH = plant height, SYPP = seed yield per plant

F_2_ seeds of each mapping population and selfed progenies of their corresponding parents were placed on Petri dishes with moist filter papers in September 2018. Up on germination, young seedlings were transferred to 2.5 L plastic pots filled with soil, in a greenhouse at the Swedish University of Agricultural Sciences (SLU, Alnarp, Sweden). Each mapping population was represented by 288 individuals, including two selfed progenies of each parent. The seedlings were grown under a 16-hour photoperiod supplied by high-pressure sodium (HPS) lamps, 21/18°C light/dark temperatures, and 60% relative humidity for two months. Then, they were transferred to a field site in Alnarp at the end of October 2018 for overwintering and further growth. The traits studied were inflorescence length (IL), plant height (PH), pod density (PD), pod shattering (PSH1 and PSH2), seed dormancy (SD), and seed yield per plant (SYPP) (Table 1; Fig. 1A). PH, PSH2, and SYPP were studied in all three mapping populations (Fig. 1A). IL was studied in MP2 and MP3, PSH1 and SD in MP1, and PD in MP3 (Fig. 1A). SD data was recorded in September 2018 while all other traits were collected during July-September 2019.

**Table 1:** List of quantitative and categorical traits targeted in this study and their descriptions

Trait	Description
Plant height (PH)^a^	Height of each plant measured in centimetre (cm) at maturity
Inflorescence length (IL)^a^	Length of the inflorescence of each plant measured at maturity: Average of the main axis and four randomly selected inflorescence branches on each plant
Pod density (PD)^a^	The ratio of the number of pods per inflorescence and inflorescence length in cm: Average of the main axis and four randomly selected inflorescence branches on each plant
Pod shattering-1 (PSH1)^a^	Twenty randomly collected dry mature pods of each genotype were analyzed in three replicates. Six 4 mm metal balls were placed together with the pods in a 50 ml grinding jar of an MM 400 mixer mill (Retsch GmbH, Haan, Germany) and shaken at a frequency of 10 Hz for a total of 20 s with data taken at 5-sec intervals (5, 10, 15, and 20 s) by counting the number of intact and half pods, with each intact pod given a value of 1 while each half pod given a value of 0.5. The average values of the four data points of the three replicates were taken for each plant.
Pod shattering-2 (PSH2)^b^	Plants were classified into four categories based on the percentage of seeds lost at full maturity after exposure to heavy rain and strong winds: Determined based on data from five randomly selected inflorescence branches. 1 = high resistance (< 10% seed loss), 2 = moderate resistance (10–30% seed loss) 3 = susceptible (30–50% seed loss), and 4 = Highly susceptible (> 50% seed loss)
Seed dormancy (SD)^b^	Seeds were classified as dormant or non-dormant based on their ability to germinate within one week after they were placed on wet filter papers in Petri dishes at room temperature:1 = Non-dormant; seeds germinated within a week, and 2 = Dormant; seeds germinated only after treatment with 700 ppm gibberellic acid (GA3)
Seed yield per plant (SYPP)^b^	Determined based on the weight of seeds harvested from each plant: 1 = low (< 5 gm), 2 = medium (5–10 gm), 3 = medium-high (11–20 gm), and 4 = high (> 20 gm)

^a^numerical and ^b^categorical values

### DNA extraction and genotyping

Leaf samples of individual genotypes of each mapping population and selfed progenies of their parents (288 individuals for each mapping population) were collected separately from 3-week-old seedlings using the BioArk™ Leaf collection kit (https://biosearchassets.blob.core.windows.net/assetsv6/guide_bioark-leaf-collection-kit.pdf) provided by LGC Biosearch Technologies (Berlin, Germany). For each sample, 10 leaf discs (6 mm diameter) were collected from each individual and placed into a well of a 96-well plate. A desiccant sachet was placed on top of each plate sealed with perforated strip caps following the guidelines of the kit, packed, and sent to LGC (Berlin, Germany) for DNA isolation and genotyping. High-quality genomic DNA was extracted using a customized Sbeadex plant kit (https://biosearch-cdn.azureedge.net/assetsv6/sbeadex-plant-data-sheet.pdf) at LGC (Berlin, Germany) and sequenced as described in [28].

### Target loci selection, SeqSNP assay design and sequencing

A total of 14,000 SNPs distributed across 4700 contigs/scaffolds (Geleta et al. 2020; https://www.ncbi.nlm.nih.gov/nuccore/WJSH01000000) were targeted for assay design in this study. 76% of them (10640 SNPs) passed the high-specificity assay design (no off-target hits allowed) and were fully covered (two oligo probes per target). Of these, 10,000 SNPs distributed across 3556 contigs/scaffolds were selected for SeqSNP-based sequencing of the three mapping populations (3 × 288 = 864 individuals). Subsequently, the SeqSNP kit was produced and contained 20,000 high-specificity oligo probes for the 10,000 SNPs, a sequencing library was constructed, and the target SNPs were sequenced. Sequencing was performed using the Illumina NextSeq 500 platform in 1 × 75 base-pair (bp) single-read mode. A total of 1.18 billion reads (1.36 million reads per sample) were obtained with an average effective target SNP coverage of 145×.

### Target SNP data filtering and *de novo* SNP discovery, and genotype assignment

The raw reads were adapter-clipped and quality-trimmed, and aligned to the reference sequence using Bowtie2 v2.2.3 [35]. A diploid SNP genotyping pipeline was set to call genotypes with a minimum coverage of eight reads per sample per locus using Freebayes v1.0.2–16 [36]. In addition to the 10,000 SNP loci originally targeted, *de novo* SNP discovery and genotype calling was also conducted in the region flanking the target loci (34 bp on either side of the original SNPs). The genotype data was filtered for SNPs with multiple alternate alleles and markers with minor allele frequency (MAF) below 1%. Individuals with missing data at more than 10% loci and markers with missing data for more than 10% individuals in each mapping population were excluded from the analysis.

### Genetic variation analysis

Pair-wise allelic differences between genotypes were calculated for each mapping population separately as well as by combining two or all three mapping populations using bi-allelic SNP markers with less than 10% missing data and a minor allele frequency of at least 1%. A principal coordinate analysis (PCoA) was conducted using the allelic difference matrix to investigate the clustering pattern of individuals in each mapping population with each other and their parents.

### Genetic linkage map construction

The filtering of genotypic data and genetic linkage map construction were performed in R-3.6.3 [37] using the package R/qtl version 1.46-2 [38] and the MSTMap algorithm [39] implemented in the package ASMap version 1.0–4 [40]. Individuals sharing greater than 90% of their alleles were considered identical. The individuals were filtered to a maximum of 16 crossovers. Markers with missing genotype information exceeding 2.5%, or showing significant segregation distortion (*p* < 0.05) after Bonferroni correction [41], and co-located markers were filtered out. The effect of all selected markers on the linkage map construction was tested with an error probability of 0.1% in the droponemarker function of R/qtl version 1.46-2 [38].

All markers with the likelihood of odds (LOD) comparison scores exceeding 3 standard deviations from the mean and all linkage groups with less than 5 markers and a length below 1 cM were removed. The final genetic map was constructed based on the maximum likelihood mapping algorithm and the Kosambi mapping function [42], with a maximal recombination frequency of 0.35, a minimal LOD score of 3, and a significance level of *p* < 1e-9, and segregation distortion of *p* < 0.1. The effect of all markers on linkage map construction was tested [38]. The genetic maps were sorted and oriented based on the corresponding SNP marker, according to the high-density genetic mapping of *Lepidium* reported earlier [10]. In cases where two linkage groups correspond to the same reference linkage group, the smaller one was added with its linkage group index not assigned to the reference map. The genetic maps were visualized in R-3.6.3 [37] using the LinkageMapView version 2.1.2 package [43].

### Quantitative trait loci (QTL) mapping

A nonparametric interval mapping, based on the non-parametric Kruskal-Wallis rank-sum test, was performed using the scanone function of R/qtl version 1.46-2 [38]. Genome-wide LOD scores were determined by running 10,000 permutations with the 99th, 95^th,^ and 90th percentiles used as thresholds for each trait. The confidence intervals for identified QTLs were determined as the approximate 95% Bayesian confidence interval in R/qtl version 1.46-2 [38]. The significance of LOD scores and their empirical *p*-values were calculated as described in DR Nyholt [44]. The percent phenotypic variance explained (PVE) by genetic variance was also calculated [45]. Additionally, a multiple QTL interval mapping (MQIM) using the mqmscan function of R/qtl was performed [38] using the markers from the Kruskal-Wallis rank-sum test (*p* < 0.05) as covariates. The window size was set to 10 cM and the genome was scanned at 1 cM interval. The covariate markers were filtered to obtain a distance of at least 10 cM from neighboring covariate markers. The genome-wide significance threshold for QTLs (LOD scores) was determined using 1,000 permutations (*p* < 0.05). QTLs detected through the interval mapping approach are designated by the prefix q, while those detected through multiple QTL interval mapping are designated by the prefix qm, followed by the mapping population index, trait abbreviation in lower case, linkage group, and serial number of the QTL found within that linkage group, followed by the mapping population index, the trait abbreviation in lower case, the linkage group, and the serial number of the QTL on that linkage group. For instance, *q1-psh2-4.1* represents the first QTL in mapping population 1 for pod shattering 2 (PSH2) in linkage group 4.

### Allelic effects of SNPs flanking significant QTLs

The effects of SNPs at loci flanking the QTLs explaining phenotype variation over 6% were investigated using the mean values of target traits for individuals with specific variants. Since the phenotypic data’s residuals were not normally distributed, a non-parametric test was applied for correlation analysis between the traits measured. The Shapiro-Wilk test was performed on quantitative phenotypic data to determine its distribution. As the data deviated from the normal distribution, all traits were analyzed using non-parametric tests. Boxplots were generated with the ggplot2 package [46]. The contribution of SNPs flanking detected QTLs for plant height and pod shattering 2 (PSH2) score was determined respectively, using the Kruskal-Wallis test and Chi-squared test in R statistical software v.4.1.2 [47].

### Identification of candidate genes within QTL regions

Taking advantage of the synteny between the *Arabidopsis thaliana* chromosomes and field cress linkage groups [10], the physical location of SNP markers flanking detected QTLs was used to search for homologous genes on the *A. thaliana* reference genome (TAIR10). The list of *A. thaliana* genes within the syntenic regions, corresponding to the QTLs identified in *L. campestre*, with their putative functional annotation was obtained using TAIR BLAST 2.9.0+.

## Results

### Phenotypic variation of the mapping populations

Correlation analysis revealed a highly significant positive correlation between plant height (PH) and seed yield per plant (SYPP) in all three mapping populations with Pearson’s correlation coefficients (r) ranging from 0.79 to 0.90 (Fig. 1B-D). Pod shattering 2 (PSH2) exhibited moderate to weak positive correlations with PH (0.14 ≤ *r* ≤ 0.44) and SYPP (0.25 ≤ *r* ≤ 0.45) across the three populations. In MP1, seed dormancy (SD) did not correlate with the other measured traits while PSH1 exhibited weak but highly significant positive correlations with PH (*r* = 0.24), PSH2 (*r* = 0.22), and SYPP (*r* = 0.24) (Fig. 1B-D). In MP2 and MP3, inflorescence length (IL) exhibited weak to moderate positive correlations with PSH2 (0.23 < *r* < 0.38), strong positive correlations with PH (0.76 < *r* < 0.8) and SYPP (0.74 < *r* < 0.78). Pod density (PD) showed moderate to strong negative correlations with the other measured traits in MP3 (-0.71 < *r* <-0.27) (Fig. 1B-D).

### SeqSNP genotyping and data filtering

Out of the 10,000 target SNPs, 9378 (94%) passed the quality criteria (≥ 8 aligned reads per sample in 85% of the samples), of which 5349 (57%) were polymorphic when the three mapping populations were considered together. However, only 849, 3786, and 2395 of these 5349 target loci were polymorphic and passed all filtering criteria within MP1, MP2, and MP3, respectively. The *de novo* SNP discovery and genotype calling resulted in 1058, 2685, and 3389 additional polymorphic SNPs passing all filtering criteria, in MP1, MP2, and MP3, respectively. As a result, the total number of polymorphic markers in the initial data set used for MP1, MP2, and MP3 analyses with 288 genotypes each was 1907, 6471, and 5784, respectively (Table 2).

The criteria set to exclude individuals with missing data at more than 10% loci and markers with missing data in more than 10% individuals resulted in the removal of 5 individuals and 109 markers from MP1, 14 individuals and 157 markers from MP2, and 15 individuals and 130 markers from MP3 (Table 2). Consequently, the number of genotypes was reduced to 283, 274, and 273 while the number of markers was reduced to 1798, 6314, and 5654 in MP1, MP2, and MP3, respectively. During the linkage map construction 9, 15, and 20 individuals were removed from MP1, MP2, and MP3, respectively, to avoid individuals sharing more than 90% of their alleles. Similarly, 984, 2867, and 1536 markers were removed due to segregation distortion, large crossover counts, and insufficient linkage to any linkage group. Hence, the final genetic linkage map was constructed using 814, 3447, and 4118 SNPs based on 274, 259, and 253 genotypes in MP1, MP2, and MP3, respectively (Table 2).

**Table 2:** Summary of the numbers of genotypes (individuals) and markers (SNP loci) used at different stages of data analysis for the three mapping populations (MP1, MP2, MP3) used in this study

Mapping population	Number of genotypes/markers in the initial dataset		Number of markers used in the final linkage map construction	
	Genotypes	Markers	Genotypes	Markers
MP1	288	1907	274	814
MP2	288	6471	259	3447
MP3	288	5784	253	4118

### The clustering pattern of F2 genotypes and their parental progenies

**Fig. 2:**
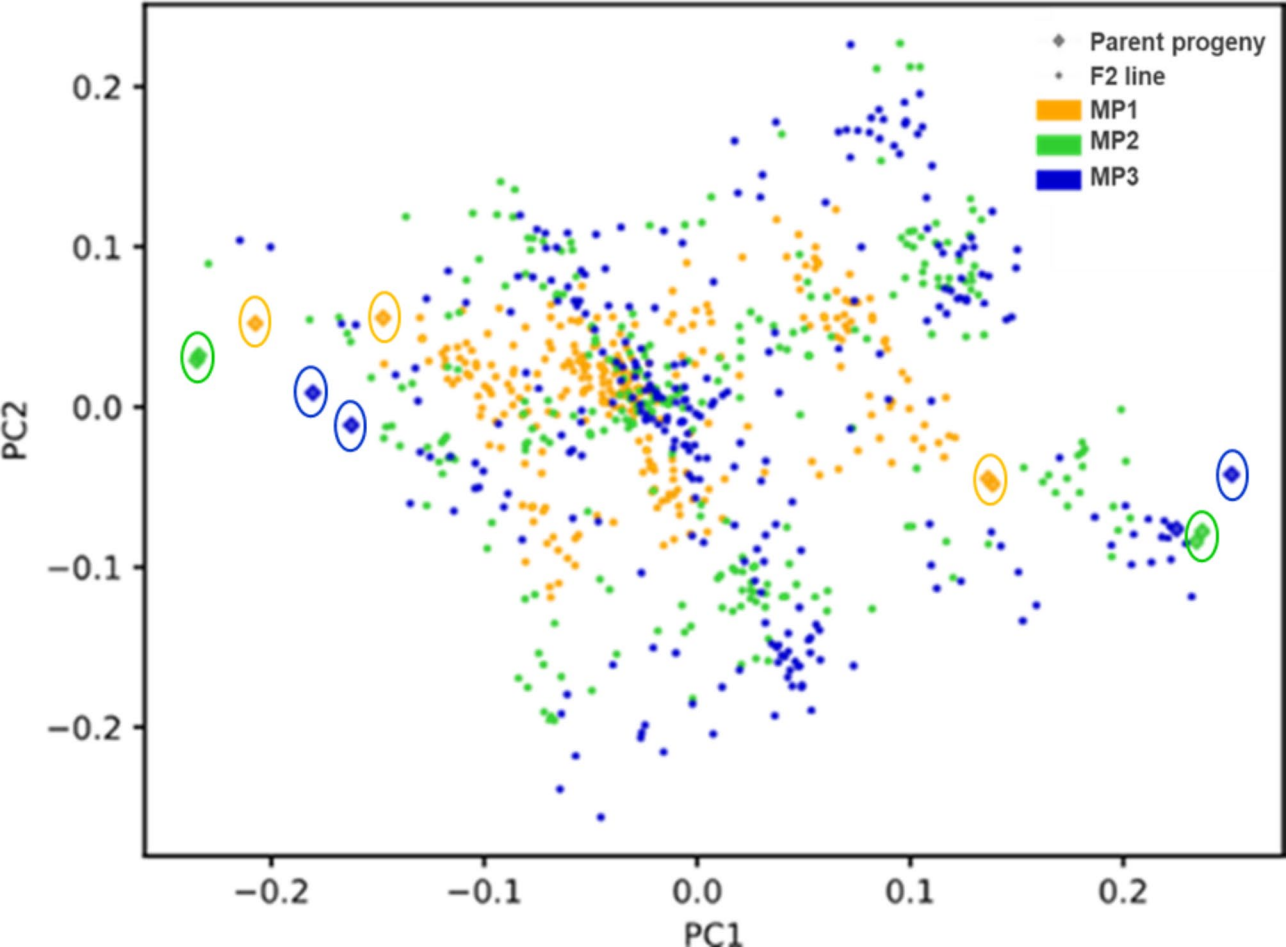
Principal coordinate analysis (PCoA) of three *L. campestre* mapping populations. Individuals of MP1, MP2, and MP3 are shown in orange, green, and blue, respectively, along with their respective selfed parental progenies. The analysis utilized 483 genome-wide bi-allelic SNPs, with less than 10% missing data and a minor allele frequency of at least 1%. The founding parents of each mapping population are represented by two selfed progenies (diamond shapes, circled)

A principal coordinate analysis (PCoA) based on allelic differences between genotype pairs across the genome-wide bi-allelic SNP loci revealed the distribution of F_2_ individuals of the three mapping populations and their respective selfed parental progenies (Fig. 2, Supplementary Fig. 1). The number of genotypes and markers used for PCoA analysis of the mapping populations separately and in combinations and the percentage of variance explained in the first three components is presented in Supplementary Table 1. The first three principal coordinates, in the analysis involving all three mapping populations, explained 24% of the total variation (Fig. 2, Supplementary Table 1).

The two selfed progenies of each founder parent used for generating the mapping populations were clustered together in this analysis. The position of these selfed progenies in the PCoA suggests a large genetic distance between the founder parents of each mapping population. The spread of F_2_ individuals in the PCoA revealed genotypes’ genetic segregation within and among the mapping populations (Fig. 2, Supplementary Fig. 1). The levels of genetic variation among individuals in MP2 and MP3 were comparable but the genetic variation within MP1 appeared to be narrower than in MP2 and MP3. The level of genetic variation within the mapping populations corresponds to the position of the selfed progenies of their parents, reflecting their genetic distance. Moreover, in each mapping population, F_2_ individuals were mostly clustered around the center and spread between the parental progenies across PC1 (Fig. 2).

### Genetic linkage map construction

**Fig. 3:**
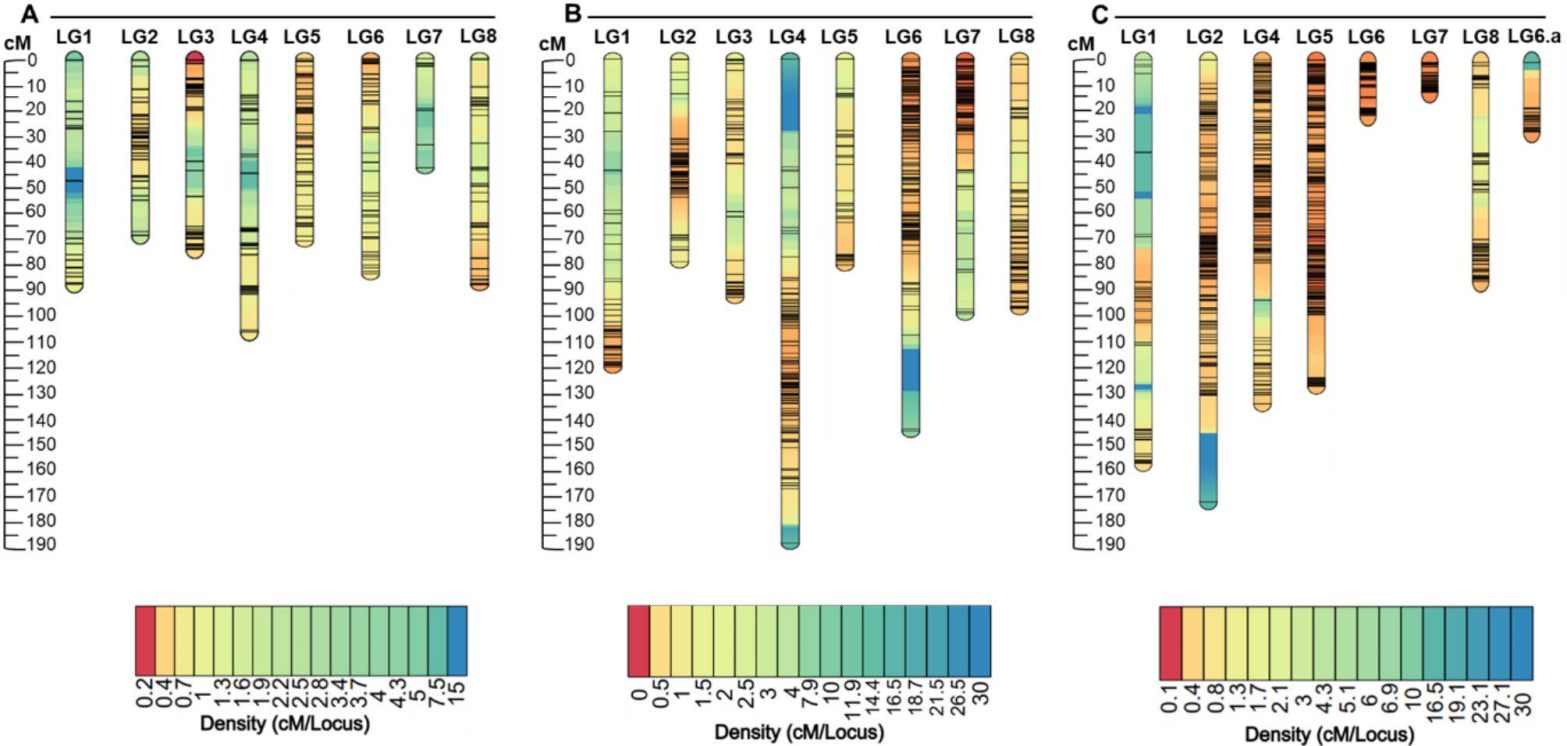
Genetic distance and marker distribution across linkage groups in three F_2_ mapping populations of field cress. Mapping population 1 (**A**), Mapping population 2 (**B**), and Mapping population 3 (**C**). The scales on the left side of each mapping population’s linkage groups are genetic distance in centimorgans (cM). The color modules below the LGs of each mapping population represent marker density in (cM/locus), from highest (red) to lowest (blue) density

In each mapping population, F_2_ individuals sharing more than 90% of their alleles, and markers with 10% missing values, distorted segregation, large crossover counts, and insufficient linkage to any linkage group were removed before the linkage map construction. The final genetic map of each mapping population comprised eight linkage groups (LGs) corresponding to the eight haploid chromosomes of *L. campestre* [10] (Fig. 3 and Supplementary Fig. 2). The total length of the LGs was 607.3 centiMorgans (cM) in MP1, 893.2 cM in MP2, and 732.7 cM in MP3 (Table 3). The largest LG was LG4 with an average map length of 140 cM while the smallest was LG5 with an average map length of 56 cM (Table 3, Supplementary Fig. 2). On average, 181 markers were mapped to MP1 LGs, 431 markers to MP2 LGs, and 514 markers to MP3 LGs. The smallest average map distance between markers was 0.065 cM (in MP3 LG1) and the largest was 2.43 cM (in MP1 LG7) (Table 3, Supplementary Fig. 2).

**Table 3:** Summary statistics of genetic linkage maps of the mapping populations (MP1, MP2, and MP3) showing the number of mapped markers, total length of linkage groups (LGs) in centiMorgans (cM), average interval between markers in cM, and the maximum interval in cM for each linkage group and mapping population

	Linkage	No of Mapped	Length	Average	Max interval
	group	markers	[cM]	interval [cM]	[cM]
**MP1**	LG1	40	85.9	2.203	19.9
	LG2	100	67.2	0.678	12.1
	LG3	132	72.7	0.555	19.5
	LG4	77	104.3	1.373	21
	LG5	167	69	0.416	4.2
	LG6	134	81.4	0.612	8.6
	LG7	18	41.3	2.430	16
	LG8	146	85.5	0.590	10.4
	**Total**	**814**	**607.3**	**1.110**	**13.96**
**MP2**	LG1	264	119.7	0.455	15.7
	LG2	423	78.8	0.187	17.4
	LG3	145	92.6	0.643	18.5
	LG4	601	188.2	0.314	41.9
	LG5	151	78.7	0.525	12.8
	LG6	800	142.3	0.178	35.9
	LG7	787	97.5	0.124	14.1
	LG8	276	95.4	0.347	11.4
	**Total**	**3447**	**893.2**	**0.750**	**20.96**
**MP3**	LG1	260	156.7	0.605	32.7
	LG2	829	171.6	0.207	41.4
	LG4	718	133.5	0.186	14.6
	LG5	1450	126.7	0.087	24.5
	LG6	315	20.5	0.065	4.1
	LG7	168	11.5	0.069	2.4
	LG8	185	85.2	0.463	25.7
	LG6a	193	27	0.140	17.8
	**Total**	**4118**	**732.7**	**0.230**	**20.4**

### QTL detection and identification

**Fig. 4:**
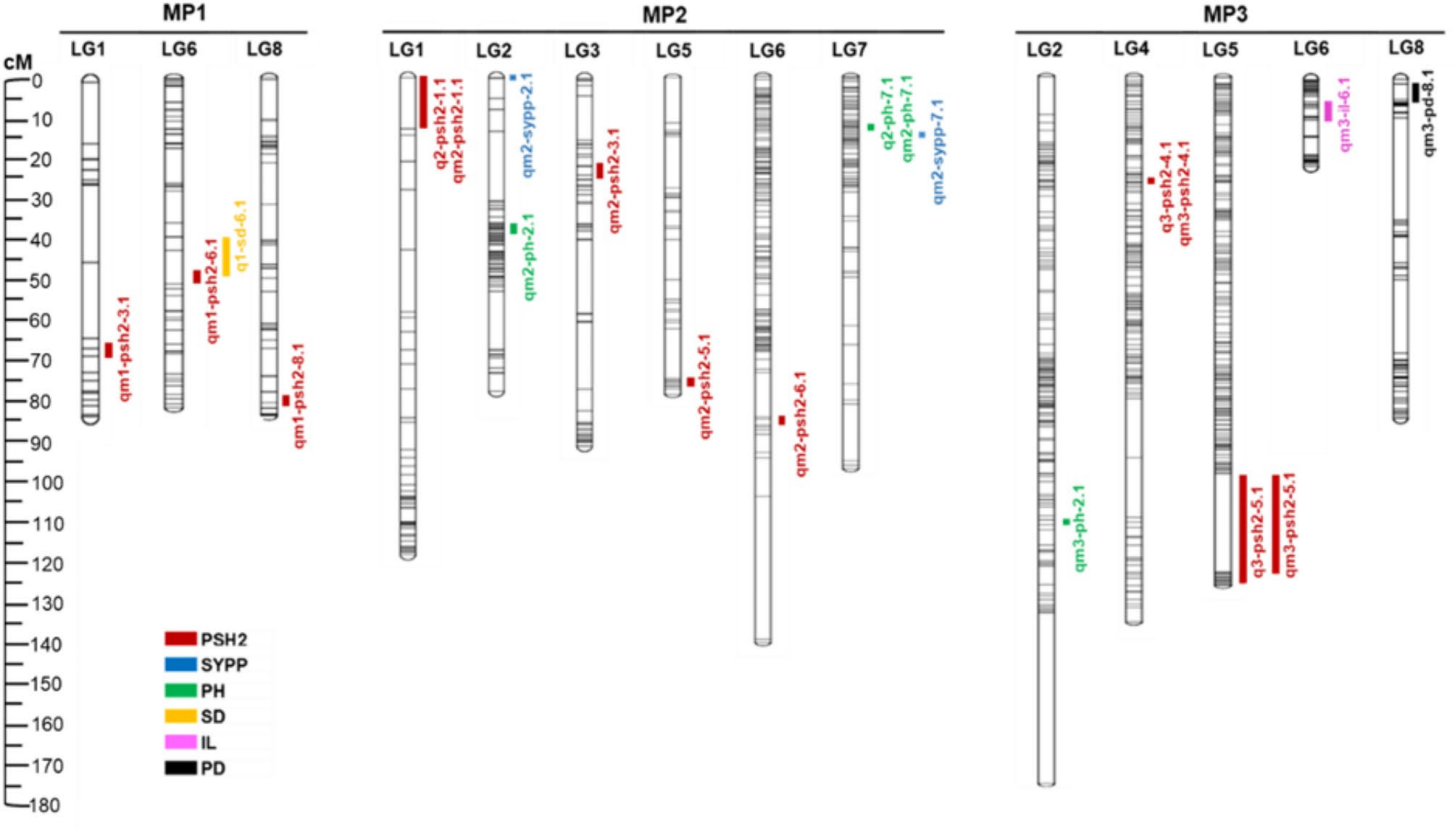
The distribution of QTLs across *L. campestre* linkage groups (LGs) in three F_2_ mapping populations (MP1, MP2, and MP3). The scale on the left indicates the LGs’genetic distance in centiMorgans (cM). Bars with different colors on the right sides of the LGs show QTL regions for different traits measured in respective mapping populations. PH: plant height, IL: inflorescence length, PD: pod density, PSH2: pod shattering, SD: seed dormancy, SYPP: seed yield per plant. A detailed description of the markers and QTLs can be found in Tables 4 and 5

Quantitative trait loci (QTL) interval and multiple interval mapping were performed for all traits measured: plant height (PH), inflorescence length (IL), pod density (PD), pod shattering (PSH1) and (PSH2), seed yield per plant (SYPP), and seed dormancy (SD). In total, 16 QTLs, linked to the measured traits, distributed across the eight linkage groups were detected in the three mapping populations (Fig. 4), using non-parametric and multiple QTL interval mapping (Supplementary Figs. 3 and 4).

The non-parametric interval mapping analysis detected four QTLs for PSH2 across four LGs and three mapping populations (Table 4; Fig. 4). The LOD scores for these QTLs ranged from 3.3 to 9.9 (Table 4). A QTL on LG8 explains 6% of the PSH2 variation in MP1, while the QTL on LG1 accounts for 5.6% of the variation in MP2. In MP3, QTLs on LG4 and LG5 explain 7.9% and 16.5% of the variation, respectively (Table 4). The narrowest QTL was on LG5 with a confidence interval of 12 cM (Table 4). One QTL for SD on LG6 was detected in MP1 with a LOD score of 3.4 and a confidence interval of 64 cM (Table 4). Moreover, one QTL for PH on LG7 was identified in MP2 with a LOD score of 4.4 and a confidence interval of 36 cM (Table 4).

The multiple QTL interval mapping detected a total of 16 QTLs distributed across all eight linkage groups (Fig. 4; Table 5). Nine of these QTLs are linked to PSH2 in three mapping populations and distributed across all LGs except LG2 and LG7. The QTL qm3-psh2-5.1 on LG5 has the shortest confidence interval length of 9.7 cM, explaining 17.9% of the phenotypic variation. Three QTLs for PH in MP2 and MP3 and two QTLs for SYPP in MP2 are in LG2 and LG7. One QTL for PD and one QTL for IL are detected on LG8 and LG6, respectively, in MP3. The average confidence interval length of the QTLs is 39 cM and explained phenotypic variances ranging from 3.8% for PH in MP2 to 17.9% for PSH2 in MP3 (Fig. 4; Table 5).

Four QTLs agree between non-parametric interval and multiple QTL interval mapping approaches: q1-psh2-8.1 and qm1-psh2-8.1, q2-psh2-1.1; qm2-psh2-1.1, q3-psh2-4.1 and qm3-psh2-4.1; and q2-ph-7.1 and qm2-ph-7.1; and q3-psh2-5.1 and qm3-psh2-5.1 (Fig. 4). Moreover, there is an overlap between q1-sd-6.1 and qm1-psh2-6.1 located on LG6 of MP1, as markers on scaffold16484_4486 have been identified as linked to both SD and PSH2 in MP1 (Table 5; Fig. 4). Furthermore, QTLs linked to PSH2 were identified in more than one mapping population on the same linkage group. On LG3, qm1-psh2-3.1 was mapped at 67 cM in MP1 and qm2-psh2-3.1 at 9 cM in MP2. On LG6, qm1-psh2-6.1 was mapped at 56 cM in MP1 and qm2-psh2-6.1 at 82 cM in MP2. Moreover, qm2-psh2-5.1 and qm3-psh2-5.1 were mapped to LG5 at 77 cM and 110 cM in MP2 and MP3, respectively.

**Table 4:** List of QTLs identified by non-parametric interval mapping in three mapping populations for PSH2, PH, and SD together with their QTL identifier, marker, linkage group (LG), position in centiMorgans (cM), confidence interval (CI) in cM, LOD score, *p*-value, and percent phenotypic variance explained. The corresponding homologous regions of the scaffolds in the *Arabidopsis thaliana* chromosomes are also shown

MP	Trait	QTL	Marker	LG	Position [cM]	CI [cM]	LOD	*P*-value	PVE [%]	Arabidopsis thaliana	
										Chr	Position
1	PSH2	q1-psh2-8.1	scaffold12832_281641	8	82.2	38-85.5	3.709	0.019	6.044	5	1,221,575–1,223,512
1	SD	q1-sd-6.1	LF: scaffold15507_247 RF: scaffold16484_4486	6	48 (LF: 42.5, RF: 50.5)	0–64	3.380	0.041	5.523	5	22,143,678–22,736,314
2	PSH2	q2-psh2-1.1	LF: scaffold33245_6144 RF: scaffold3772_160	1	6.0 (LF: 0, RF: 12.8)	0–16	3.268	0.071	5.646	1	19,846,981–8,398,783
2	PH	q2-ph-7.1	scaffold7796_112581	7	13.1	2–38	4.366	0.005	7.469	4	15,798,961–15,800,595
3	PSH2	q3-psh2-4.1	scaffold24113_3118	4	24.9	13–100	4.530	0.004	7.915	2	14,751,580–14,753,261
		q3-psh2-5.1	LF: scaffold5167_110773 RF: scaffold2511_123	5	110 (LF: 98.9, RF: 123.4)	104–116	9.914	< 0.0001	16.511	3	19119736–21385955)

PH: plant height, PSH2: pod shattering 2, SD: seed dormancy, PVE: percent phenotypic variance explained, RF: right flanking marker to LOD peak, LF left flanking marker to LOD peak, MP: mapping population; Chr: Chromosome; LG: Linkage group

**Table 5:** List of QTLs detected by multiple QTL interval mapping in three mapping populations for PSH2, PH and SD together with their QTL identifier, marker, linkage group (LG), position in centiMorgans (cM), confidence interval (CI) in cM, LOD score, *p*-value, and percent phenotypic variance explained. The corresponding homologous regions of the scaffolds in the *Arabidopsis thaliana* chromosomes are also shown

MP	trait	QTL	Marker	LG	Position [cM]	CI [cM]	LOD	*P*-value	PVE [%]	Arabidopsis thaliana	
										Chr	position
1	PSH2	qm1-psh2-3.1	LF: scaffold20235_300 RF: scaffold15372	3	67 (LF: 65.8, RF: 68.4)	59–75	3.338	0.006	5.456	1	4,123,932–3,835,472
		qm1-psh2-6.1	LF: scaffold16484_4486 RF: C1433542_665	6	56 (LF: 55.6, RF: 56.9)	53–59	4.286	0.001	6.951	5	22,737,029–22,933,641
		qm1-psh2-8.1	LF: scaffold5384_151159 RF: scaffold12832_281641	8	82 (LF: 80, RF: 82.6)	73-86.6	4.038	0.002	6.562	5	LF: 1,429,163–1,200,705
2	PSH2	qm2-psh2-1.1	LF: scaffold33245_6144 RF: scaffold3772_160	1	9 (LF: 0, RF: 12.8)	3–17	3.027	0.012	5.241	1	19,846,981–8,398,938
		qm2-psh2-3.1	LF: scaffold32066_20826 RF: scaffold32066_20834	3	24 (LF: 22.2, RF: 24.4)	19–37	2.809	0.019	4.871	4	4,672,786–3,751,698
		qm2-psh2-5.1	LF: C1402956_453 RF: scaffold22013_3008	5	77 (LF: 76.8, RF: 77.3)	0-79.7	2.340	0.068	4.076	3	22,507,794–23,038,898-
		qm2-psh2-6.1	LF: scaffold15787_4396 RF: scaffold3533_323	6	82 (LF: 81.4, RF: 85.1)	13.5-122.7	2.352	0.064	4.095	5	21,322,349–21,642,352
2	SYPP	qm2-sypp-2.1	scaffold33325_2989	2	0	0-77.2	2.298	0.070	4.003	1	23,452,106–23,588,351
		qm2-sypp-7.1	LF: scaffold7796_123473 RF: scaffold7796_174164	7	14 (LF: 13.9, RF: 14.3)	2.6–47	3.407	0.007	5.879	4	15,775,801–15,767,162
2	PH	qm2-ph-2.1	LF: scaffold24587_13489 RF: scaffold25785_23389	2	36 (LF: 35.6, RF: 36.3)	26–41.0	2.418	0.055	4.208	2	3,810,443–6,532,297
		qm2-ph-7.1	LF: scaffold7796_147189 RF: scaffold7796_112581	7	13 (LF: 12.7, RF: 13.1)	2.6–17	5.420	< 0.0001	9.188	4	15,794,341–15,790,447
3	PSH2	qm3-psh2-4.1	LF: scaffold24113_3118 RF: scaffold30019_122	4	25 (LF: 24.9, RF: 25.7)	10-73.6	3.407	0.005	6.013	2	14,752,538–14,694,270
		qm3-psh2-5.1	LF: scaffold5167_110773 RF: scaffold2511_122	5	110 (LF: 98.9, RF: 123.4)	104–116	10.828	< 0.0001	17.888	3	19,119,736–21,385,955
3	PD	qm3-pd-8.1	LF: scaffold14264_3808 RF: scaffold17154_2876	8	4 (LF: 3.4, RF: 4.2)	1–34	2.666	0.019	4.737	5	19,049,624–18,906,452
3	IL	qm3-il-6.1	LF: scaffold630_2312 RF: C1377784_867	6	8 (LF: 7.5, RF: 9.5)	0–16	2.563	0.023	4.557	1,5	18,566,603–18,568,225
3	PH	qm3-ph-2.1	LF: scaffold26748_5062 RF: scaffold20495_577	2	110 (LF: 109.2, RF: 110.2)	13-135.3	2.133	0.059	3.808	1	26,590,312–26,645,931

PH: plant height, IL: inflorescence length, PD: pod density, PSH2: pod shattering, SYPP: seed yield per plant, RF: right flanking marker to LOD peak, LF left flanking marker to LOD peak, MP: mapping population; Chr: Chromosome; LG: Linkage group

### SNP variant effect on the targeted traits

**Fig. 5:**
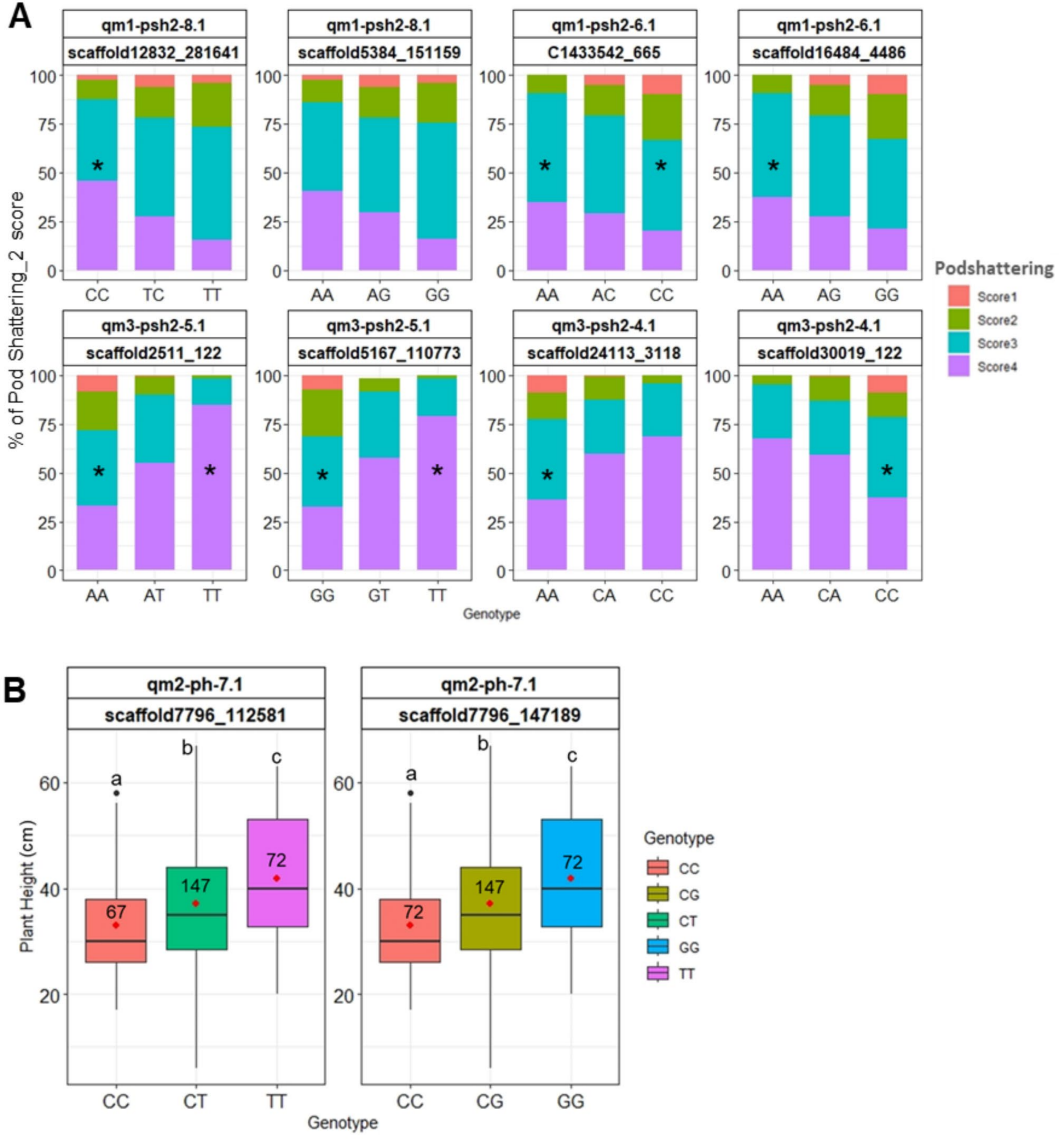
Effect of significant SNPs flanking QTLs linked to (**A**) pod shattering (PSH2) and (**B**) plant height (PH) in three F_2_ mapping populations (MPs) of *L. campestre*. Only QTLs explaining phenotypic variation over 6% are presented. QTLs for PSH2 qm1-psh2-8.1 and qm1-psh2-6.1 were identified in MP1, and qm3-psh2-5.1 and qm3-psh2-4.1 in MP3. A QTL for PH, qm2.ph-7.1, was identified in MP-2. In (**A**) PSH2 scores range from 1 (most resistant) to 4 (most susceptible) and *indicates their significance (*P* < 0.05) chi-squared (χ^2^) distribution for each SNP marker flanking the significant QTL. (**B**) Plant height values for homozygous and heterozygous genotypes at the bi-allelic loci flanking the significant QTLs. The red dots indicate the mean plant height for each genotype group, while numbers within boxes represent the count of F_2_ individuals with specific genotypes at the corresponding SNP locus. Genotypes represented with different small letters were significantly different in plant height (*P* < 0.05)

The variation in phenotypic values of homozygous and heterozygous genotypes at the bi-allelic SNP loci flanking the QTLs is presented in Fig. 5. In general, the result indicates that the homozygous genotypes had either the highest or the lowest mean phenotypic values while the heterozygous genotypes had intermediate phenotype values. In all mapping populations, the genotypic ratio at all significant SNP loci was 1:2:1, which is an expected segregation ratio in the F_2_ population obtained by selfing heterozygous F1 individuals (Fig. 5). Compared to their homozygous counterparts, individuals heterozygous for all the markers flanking the significant QTLs showed intermediate PSH2 scores (Fig. 5A) and plant height (Fig. 5B).

The chi-square (χ^2^) distribution of the PSH2 trait for each variant of the SNP markers flanking the most significant QTLs is presented in Supplementary Table 2. All variants of the flanking markers with significant χ^2^ probability distribution (*P* < 0.05) of the PSH2 scores were homozygotes (Fig. 5A). In MP1, 10% of the individuals with qm1-psh2-6.1 flanked by CC (C1433542_665) and GG (scaffold16484_4486) and 6% of individuals with qm1-psh2-8.1 flanked by the heterozygous TC (scaffold12832_281641) and AG (scaffold5384_151159) were resistant to pod shattering (score 1). In this population, 35% of the individuals with qm1-psh2-6.1 region flanked CC and AA or qm1-psh2-8.1 flanked by AA were susceptible to pod shattering (score 3 and 4) (Fig. 5A). In MP3, 8% of the individuals homozygous for markers flanking qm3-psh2-4.1 AA (scaffold24113_3118) and CC (scaffold30019_122) and for qm3-psh2-5.1 AA (scaffold2511_122) and GG (scaffold5167_110773) were resistant to pod shattering (score 1). When the qm3-psh2-5.1 is flanked by homozygous TT, 80% of the individuals in MP3 were susceptible to pod shattering (score 4) (Fig. 5A).

Individuals homozygous for scaffold7796_147189 (GG) and scaffold7796_112581 (TT), flanking qm2-ph-7.1 (PVE 9.2%, Tables 4 and 5), were taller compared to homozygous individuals (CC) or heterozygous individuals for these flanking markers (*P* < 0.05) (Fig. 5B).

### Potential candidate genes located in the major QTL for pod shattering

Both non-parametric interval mapping and multiple interval mapping uncovered a major QTL for pod shattering (named q3-psh2-5.1 and qm3-psh2-5.1, respectively) that explained 17% of the phenotypic variation (Tables 3 and 4). This QTL is located on the LG5 and corresponds to the homologous region covering 2.2 million base pairs on chromosome 3 of *A. thaliana* (Table 5). The syntenic block of *A. thaliana* reference genome TAIR10 that corresponds to this QTL contains 770 candidate genes (Supplementary Table 3). These include genes reported to be involved in growth and development and plant response to biotic and abiotic stress (Supplementary Table 4). Genes related to cellulose, pectin, and lignin biosynthesis (AT3G53520, AT3G56960, AT3G52600, AT3G57130, AT3G54260, AT3G57420, AT3G56000), which are involved in cell wall deposition and possibly affect pod shattering are among these in the list. There were also genes encoding for ADPG1, a polygalacturonase protein involved in silique and anther dehiscence, and ABCG half-transporters that are required for the synthesis of an effective suberin barrier known to prevent water loss from plant tissues including seeds. Several other genes encoding for domain transcription factors (AP2, MADS-box, bZIP domain, and C2HC zinc finger) and other proteins (calmodulin, Fasciclin-like arabinogalactan protein, INO80, GRF, MMZ4/UEV1D, NPU) related to flowering, pollen, anther, seed, and silique development were also identified. The list also includes genes encoding PLC5, C2H2, DREB2A, and E3 ubiquitin ligase proteins, which are linked to plant response to drought.

## Discussion

In recent years, the domestication of lesser-known plant species has emerged as a valuable strategy to diversify agriculture and support resilience against environmental stresses. Unlike traditional crops, which often face genetic bottlenecks due to extensive breeding, these newer species offer untapped genetic resources. By targeting specific traits for adaptation and sustainability, the domestication of these species can meet the growing demands for crops that are both high-yielding and resilient to changing climates, reinforcing the importance of expanding agricultural biodiversity. Domesticating new plant species with distinct traits not found in widely cultivated crops is crucial for addressing the growing demands for high-yield, high-quality crops. It also enhances the resilience of cropping systems, and sustainably mitigates the impacts of changing climate conditions. Currently, there are a few other plant species, such as field pennycress (*Thlaspi arvense*) and silphium (*Silphium integrifolium*) targeted for domestication [48–50]. However, domestication is a protracted process and can lead to a loss of fitness present in the wild species [1], which hampers the productivity of cultivated crops under low-input conditions. Developing genomic resources for crops under domestication is essential for understanding and maintaining genetic diversity, efficiently utilizing the gene pool, identifying closely related wild species as a source of desirable traits, fast-tracking the domestication process, and further improving the domesticated crop.

This study was aimed to develop genomic tools for *L. campestre*, a species under domestication as a novel catch crop and oil crop for industrial purposes [3, 4]. By sequencing and phenotyping three F_2_ bi-parental *L. campestre* populations we constructed genetic linkage maps and identified QTLs linked to key domestication traits. Genetic linkage maps have previously been constructed [10] and QTLs linked to domestication-related traits have been identified in *L. campestre* [24]. However, these previous studies used mapping population based on interspecific hybrids between *L. campestre* and close relative *L. heterophyllum.* In contrast, this study contributes to understanding the recombination between genotypes of *L.campestre*, and the genetic linkage map presented here provides crucial insights into the genetic regulation of the targeted traits in *L. campestre*. Although bi-parental population-based studies are limited by only capturing recombination between the founder parents, they are simple and powerful tools to detect QTLs and dissecting the genetic basis of complex traits [51]. But it is worth noting that in mapping population MP3, there was no linkage group (LG) constructed for chromosome 3 (LG3), indicating a lack of sufficient DNA sequence variation (polymorphisms) to map genetic markers for this chromosome. Additionally, chromosome 6 (LG6) in MP3 showed enough differences in genetic data that it was split into two separate linkage groups, designated as LG6 and LG6a. This reflects challenges in constructing a continuous genetic map for those specific chromosomes using the bi-parental mapping populations. The distribution of individuals on the PCoA plot along with the observed phenotypic variation, and the 1:2:1 segregation indicates that the alleles from the founder parents have been successfully assayed and represented in the three *L. campestre* mapping populations investigated.

Detecting QTLs linked with desirable traits is essential for targeting genomic regions and genes to improve agronomic traits through new breeding techniques such as transgenics and gene editing [15–17]. Likewise, it provides a set of markers for developing and applying markers-assisted selection to accelerate the breeding process [25–27]. Previously C Hammenhag, GV Saripella, R Ortiz and M Geleta [24] detected QTLs linked to the number of stems per plant, stem growth orientation, plant height, and perenniality in *L. campestre*. Using QTL interval and multiple mapping approaches we have in this study identified additional QTLs linked to six domestication-targeted traits: pod shattering, pod density, plant height, seed yield per plant, seed dormancy and inflorescence length. These traits are known to be regulated by multiple genes and QTLs [20–22]. In total, nine significant QTLs for PSH2 were identified on several LGs in all three populations, with varying levels of effect on the phenotype. The mapping populations were developed based on different founding parents. The number of markers, and their heterozygosity levels, used for QTL analysis varies across these populations. It should be noted, however, that some of the QTLs linked to PSH2 were identified on the same LGs in more than one mapping population. Further research using a chromosome-level reference genome will shed light on whether they refer to the same genes. The result shows that pod shattering, which is a classical ‘domestication syndrome’ trait [1], is regulated by multiple QTLs located on different chromosomes. Indeed, studies in *Brassicaceae* show that several QTLs can contribute to pod-shattering resistance [52–54] although dominant genes contributing to pod shattering resistance have also been identified [55].

The major QTL for PSH2, qm3-psh2-5.1/q3-psh2-5.1 exhibited a LOD score greater than 9 and PVE greater than 16%. This QTL region contains candidate genes with putative functions for flower, pollen, seed, and silique development. One of the genes, AT3G57510, encodes the Arabidopsis Dehiscence Zone Polygalacturonase 1 (ADPG1) protein, which is normally expressed during anther and silique dehiscence (ADPG1) and is known to be involved in these dehiscence processes [56]. Moreover, genes within this QTLs are putatively involved in cellulose and lignin biosynthesis, crucial for cell wall deposition and strongly linked to pod shattering resistance [57]. The results suggest that the QTL detected here could be a hotspot for identifying and targeting genes to improve pod shattering resistance in *L. campestre*.

A QTL for plant height (PH) was identified in MP2 on LG7 (qm2-ph-2.1/ q2-ph-7.1), which explains 9% of the total observed variation. Previously, a QTL for plant height on LG7 of *L. campestre* was identified, also explaining 9% of the phenotypic variation [24]. However, in [24] the QTL was located at 48 cM, whereas in this study qm2-ph-2.1/q2-ph-7.1 was detected at 13 cM. It is worth noting, as mentioned above, that unlike in the current study [24], used a mapping population derived from interspecies hybridization which had a lower marker density compared to the 3,447 SNP markers used in MP2, in this study. Interestingly, qm2-ph-2.1/q2-ph-7.1 was adjacent to the major QTL for seed yield per plant (SYPP) qm2-sypp-7.1, in MP2, explaining the strong correlation observed between PH and SYPP traits across all three mapping populations. Furthermore, the QTL qm3-il-6.1 for inflorescence length (IL), which showed a strong positive correlation with PH and SYPP in both MP2 and MP3, was detected in MP3. A strong correlation between PH, SYPP and IL has been reported previously in *Brassica* species [58–60], with the relationship among these traits further supported by the colocalization of their QTLs [60–62]. Similarly, the QTL qm3-pd-8.1 for pod density (PD), which showed a negative correlation with PH, SYPP, and IL, was detected in MP3. In MP1, QTL q1-sd-6.1 was identified for seed dormancy (SD), a trait crucial for seed germination and seedling establishment and, subsequently yield and quality. Since SD is a critical trait for domestication this finding provides important genetic insight to understanding its regulation and the accurate selection of non-dormant genotypes. However, the significant positive correlation should be verified by evaluating Lepidium populations under different environments before plant height is considered a major phenotypic marker for selecting high-yielding genotypes, together with other traits, such as inflorescence length and pod density. This will shed light on the genetic and environmental factors contributing to the relationship between these traits.

We further investigated the effect of the SNP markers flanking the most significant QTLs with PVE greater than 6%. The observed pattern for pod shattering resistance and plant height is consistent with the concept of additive genetic effects, where the presence of different alleles can result in intermediate phenotypes compared to the extremes observed in the homozygous genotype. Alleles flanking the locus qm1-psh2-6.1, qm1-psh2-8.1, qm3-psh2-4.1, q3-psh2-5.1/ qm3-psh2-5.1 and are linked to lower pod shattering can be considered as sources of resistance and for combining desirable alleles and improve this indispensable trait. Moreover, in MP2, individuals with the alleles GG for scaffold7796_147189 and TT for scaffold7796_112581 (flanking qm2-ph-7.1) are taller than genotypes with other alleles. Identification of these SNPs and their contribution to the phenotypic variability will significantly enhance practical breeding efforts.

## Conclusion

Here, we used three F_2_ bi-parental mapping populations to demonstrate that plant height and inflorescence length are key traits for selecting *L. campestre* genotypes with higher seed yield per plant and reduced pod shattering. The comprehensive linkage map constructed in this study along with the QTLs identified for key agronomic traits (PSH2, PH, SYPP, IL, PD, and SD), the list of candidate genes within these QTL regions, and insight into potential roles of SNPs flanking these QTLs form a valuable genomic resource. Together, they provide a foundation for fast-tracking the domestication of *L. campestre* by facilitating targeted selection and improved trait management. Genetic markers are crucial for understanding genetic diversity, conducting studies such as genome-wide association study (GWAS), and effectively utilizing and preserving valuable diversity. This study’s findings highlight critical genomic regions and candidate genes that can be targeted for developing marker-assisted strategies and employing new breeding tools such as gene editing for speeding up the domestication of *L. campestre*. However, constructing a higher-resolution genetic map with enhanced prediction accuracy, genome assembly, and annotation should be complemented by advanced sequencing techniques. These mapping populations should be phenotyped under multi-field conditions to address QTL-by-environment interactions (QEI) and confirm trait heritability ensuring wider application of the genetic markers. Beyond characterization of the key agronomic traits, the mapping should be extended for response to biotic and abiotic stresses, as well as oil yield and quality to lay the foundation for continuous improvement of domesticated versions of *L. campestre*, enhancing its adaptability and commercial potential in varied agricultural contexts.

## Electronic supplementary material

Below is the link to the electronic supplementary material.

**Supplementary Material 1**: **Supplementary Table 1.** Genetic population difference analysis and explained variance of the first three components of the PCoA. **Supplementary Table 2.** Chi-square test for the probability distribution of pod shattering 2 (PSH2) score for each marker flanking the significant QTLs. **Supplementary Table 3.** QTLs detected using Multiple QTL mapping analysis and the list of genes identified in Arabidopsis synteny regions corresponding to QTL regions in field cress. **Supplementary Table 4.** List of annotated genes found in Arabidopsis within the synteny region for the QTL significantly linked to pod-shattering (q3-psh2-5.1 /qm3-psh2-5.1), explaining 17% of the total variation.

**Supplementary Material 2**: **Supplementary Fig. 1.** PCoA plot based on SNP allele displaying genetic differences and similarities among the progenies and their respective founding parents of three field cress mapping populations MPs): MP1 MP2 and MP3.

**Supplementary Material 3**: **Supplementary Fig.2.** Genetic linkage of three bi-parental mapping populations of field cress.

**Supplementary Material 4**: **Supplementary Fig.3.** Multiple QTL interval mapping in three bi-parental mapping populations of field cress. (A) Mapping population 1, MP1; (B) Mapping population 2, MP2; (C) Mapping population 3, MP3. Plant height (PH), pod shattering (PSH1 and PSH2), seed yield per plant (SYPP), inflorescence length (IL), and seed dormancy (SD). The dotted lines indicate LOD score thresholds (red: *P* = 0.1, orange: *P* = 0.05, green: *P* = 0.01).

**Supplementary Material 5**: **Supplementary Fig.4.** QTL interval mapping in three bi-parental mapping populations of field cress. (A) Mapping population 1, MP1; (B) Mapping population 2, MP2; (C) Mapping population 3, MP3. Plant height (PH), pod shattering (PSH1 and PSH2), seed yield per plant (SYPP), inflorescence length (IL), and seed dormancy (SD). The dotted lines indicate LOD score thresholds (red: *P* = 0.1, orange: *P* = 0.05, green: *P* = 0.01).

## Data Availability

Availability of data: All important data are included in the submitted manuscript. The datasets generated and/or analysed during the current study are available in the Availability of data: All the important data are included in the submitted manuscript. The datasets generated and/or analysed during the current study are available in the Figshare repository, https://figshare.com/s/7456c95a2bbf747058d5 (Doi: 10.6084/m9.figshare.27901644). Availability of materials: The list of all mapping populations and their genotyping and phenotype data is readily available upon request. Seeds can be shared upon request in compliance with national and international guidelines for exchanging genetic materials.
