# Supplementary figures and images for: Whole-genome mapping reveals QTLs linked to key agronomic traits in bi-parental populations of field cress (Lepidium campestre)
Source: BMC Plant Biol. 2025 Feb 24;25:246. doi: 10.1186/s12870-025-06197-3 (PMC11849345; doi:10.1186/s12870-025-06197-3)

# Mapping Population 1

LG1

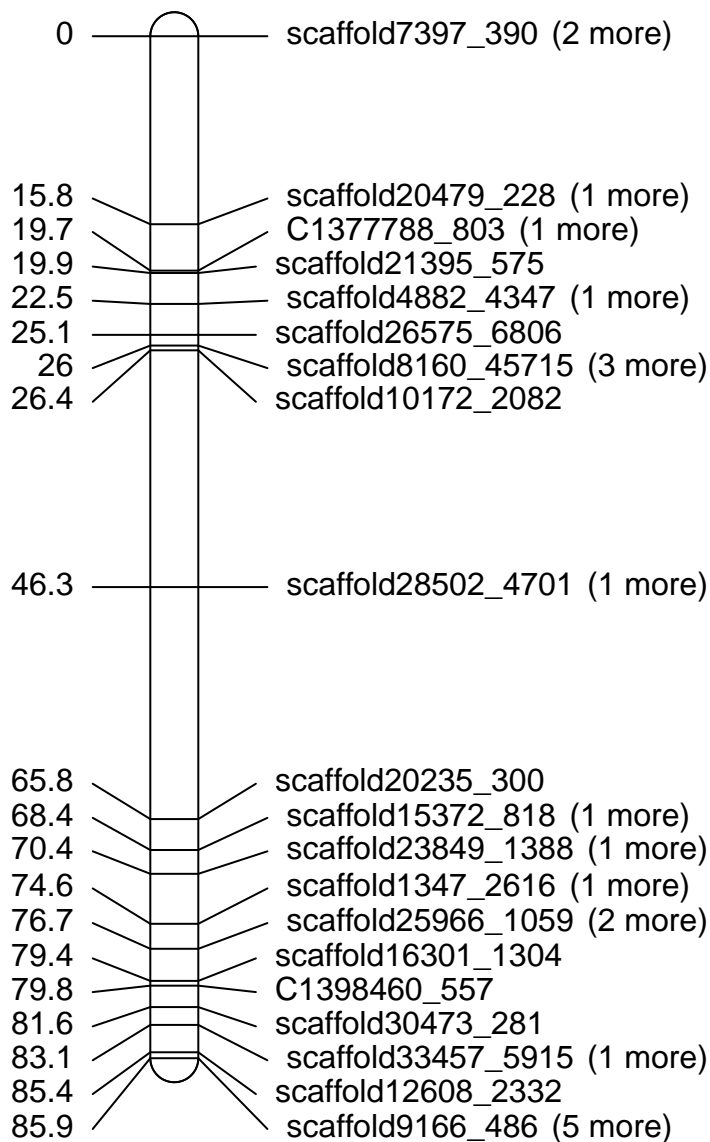

LG2

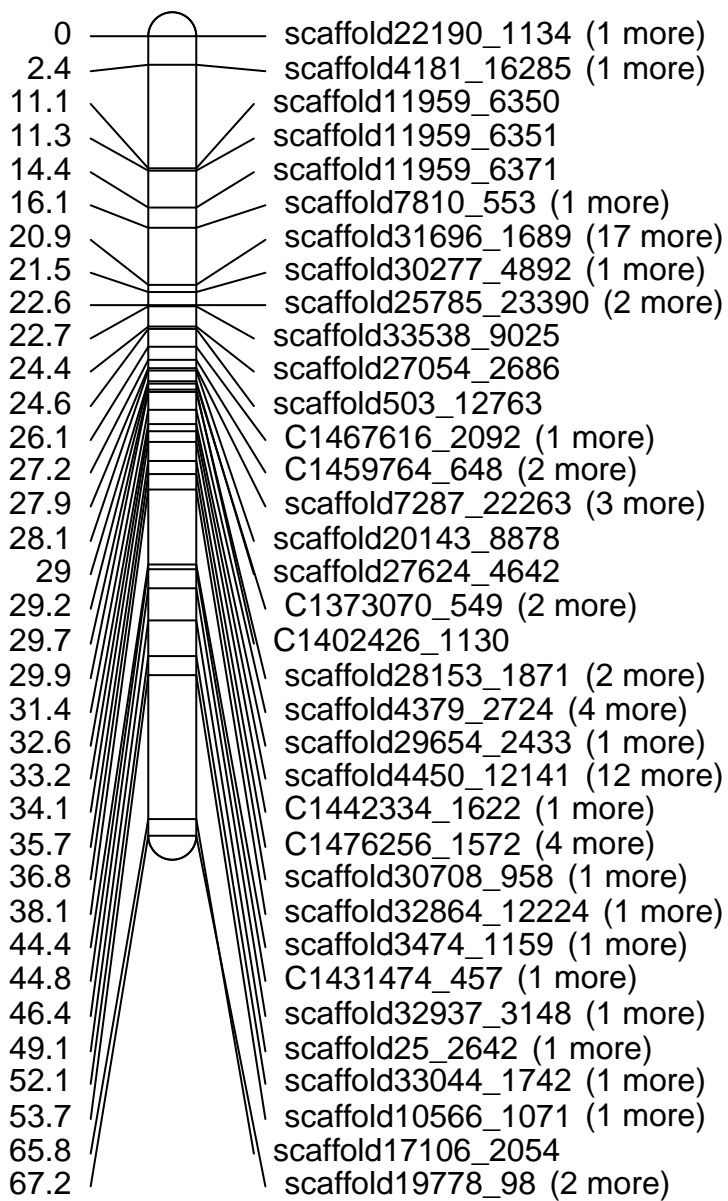

LG3

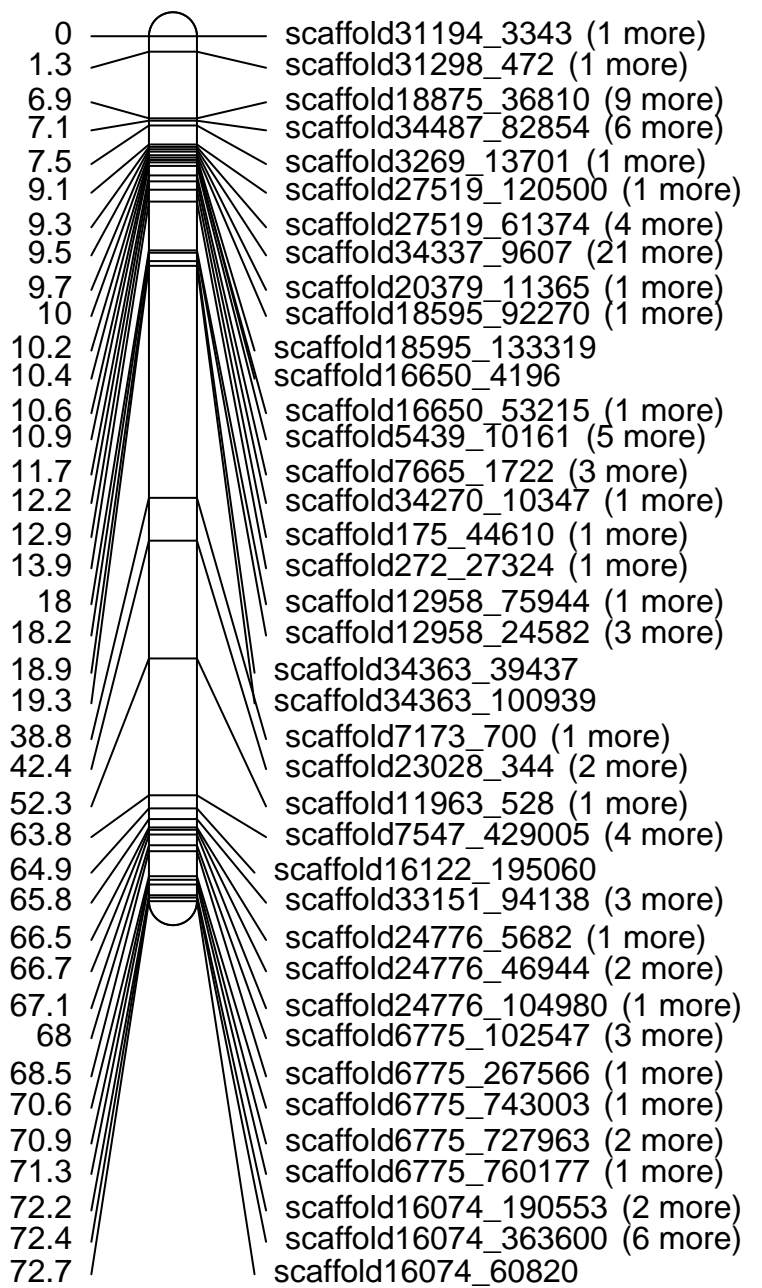

LG4

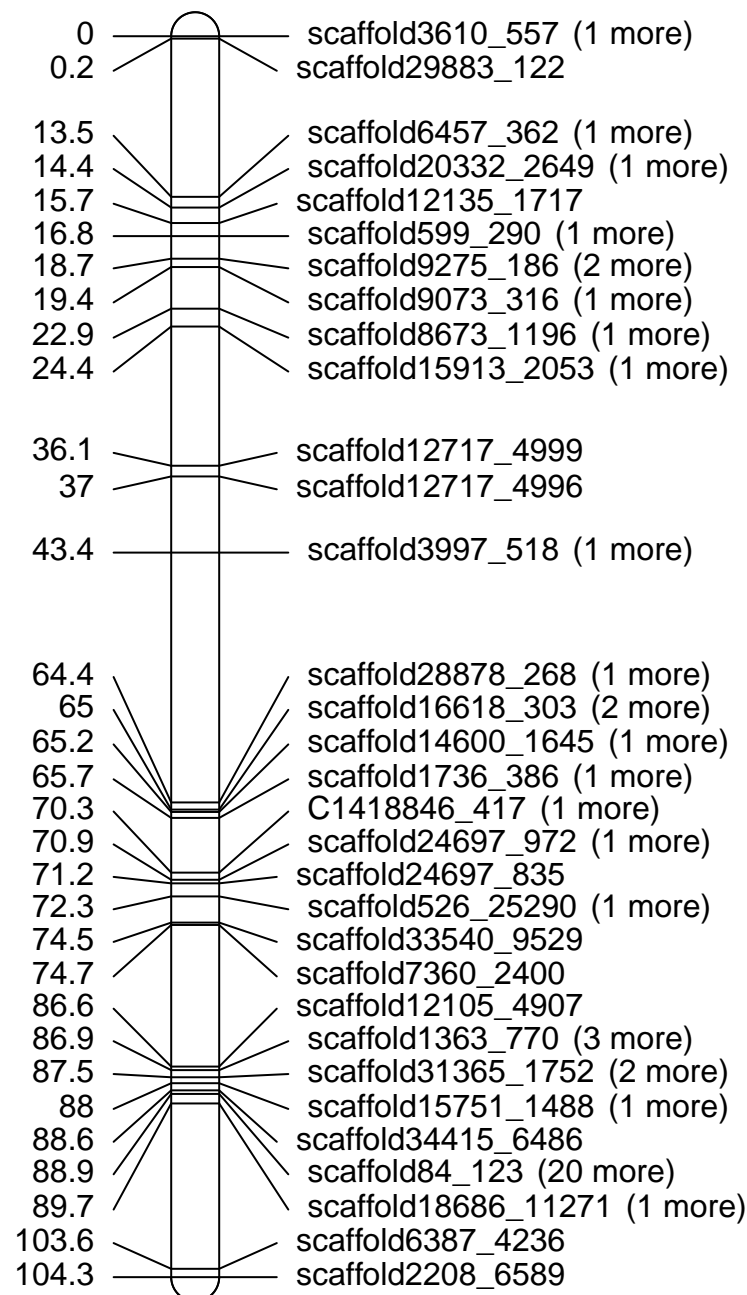

LG5

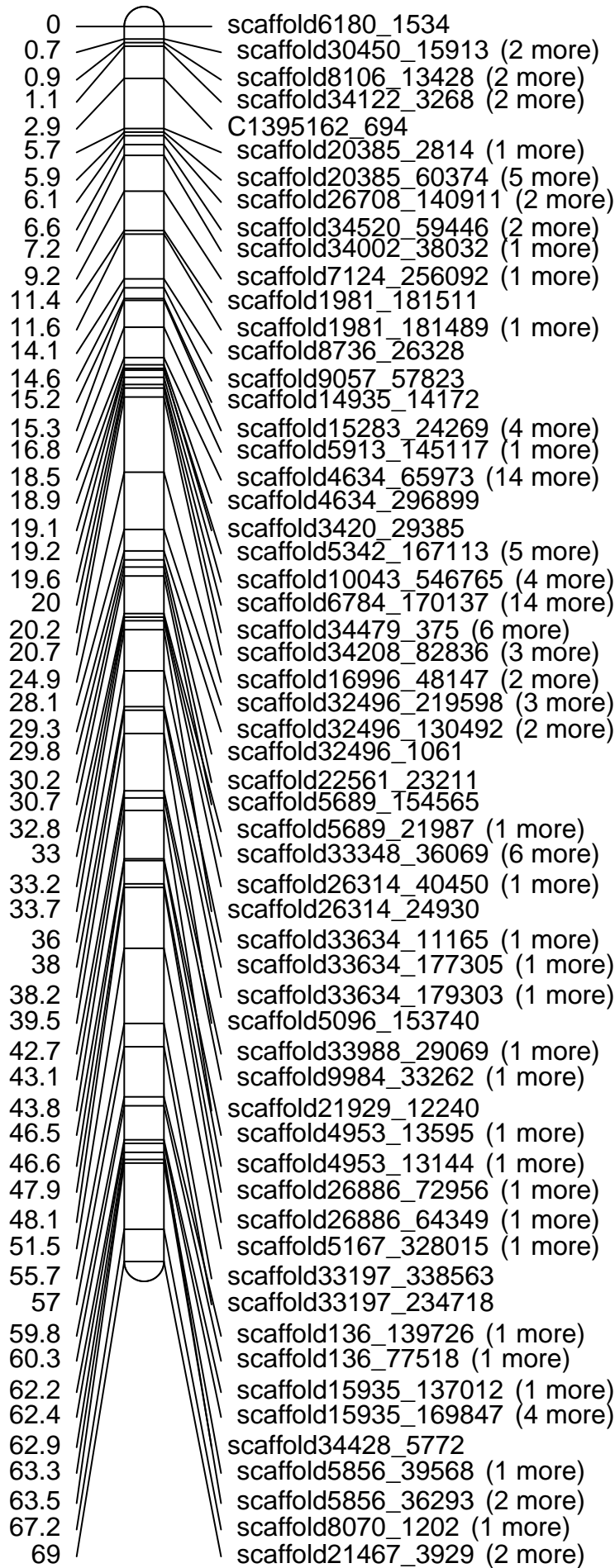

LG6

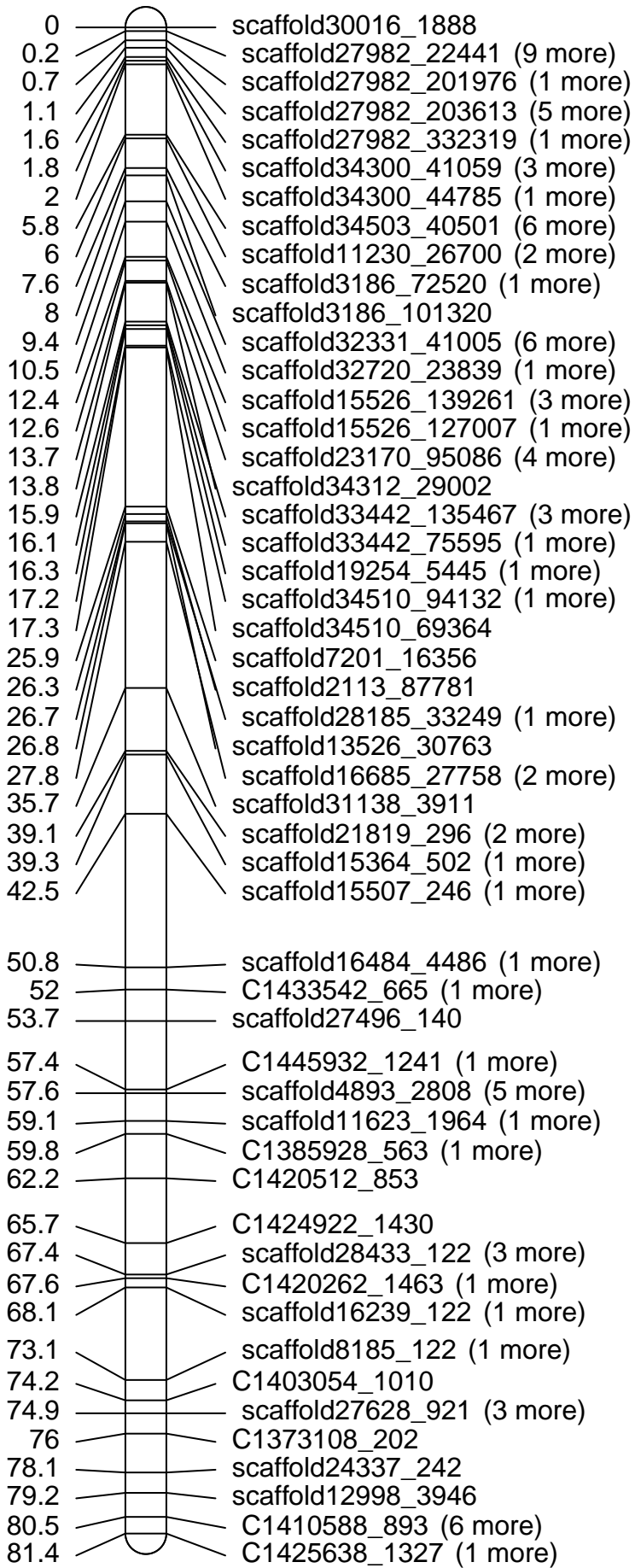

LG7

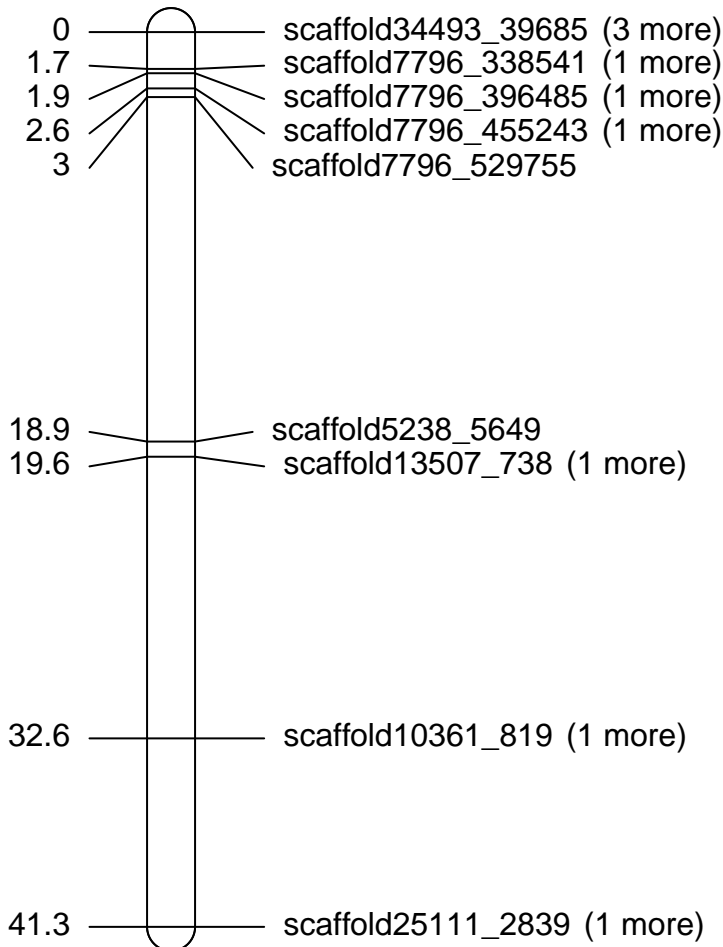

LG8

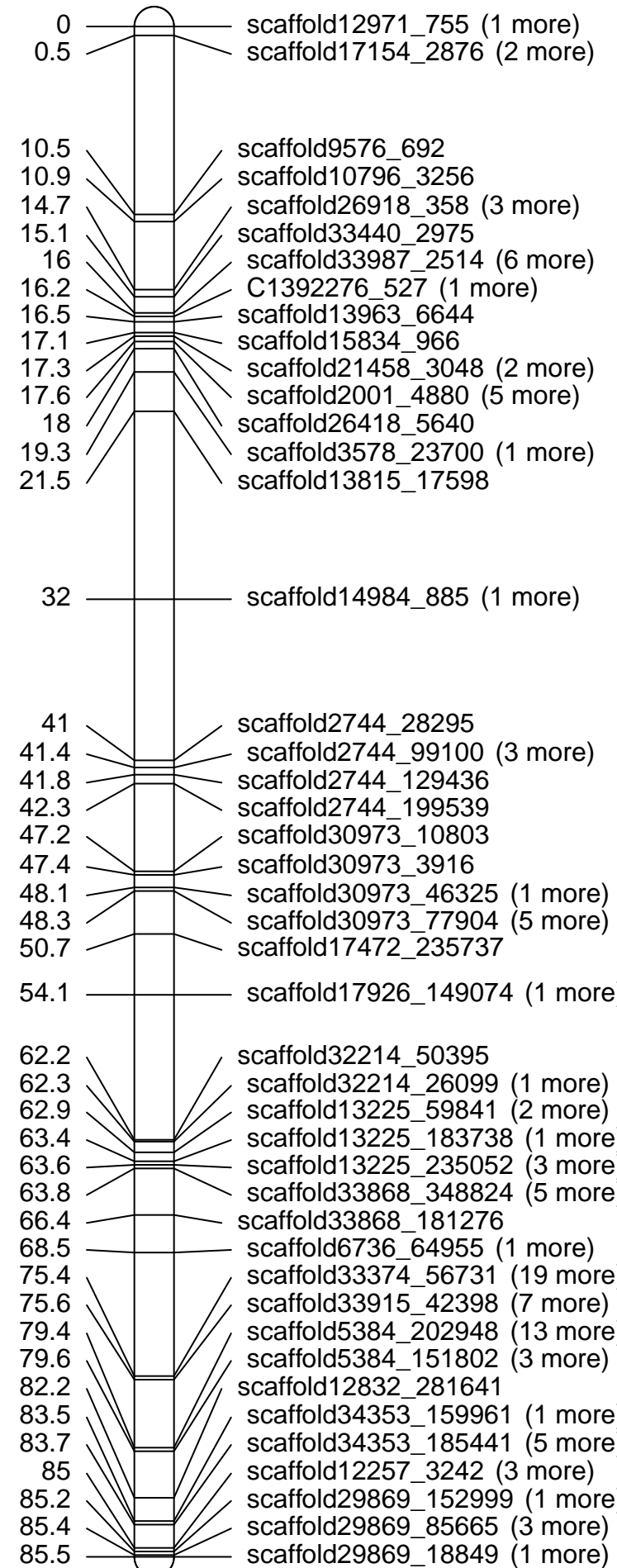

Mapping Population 2

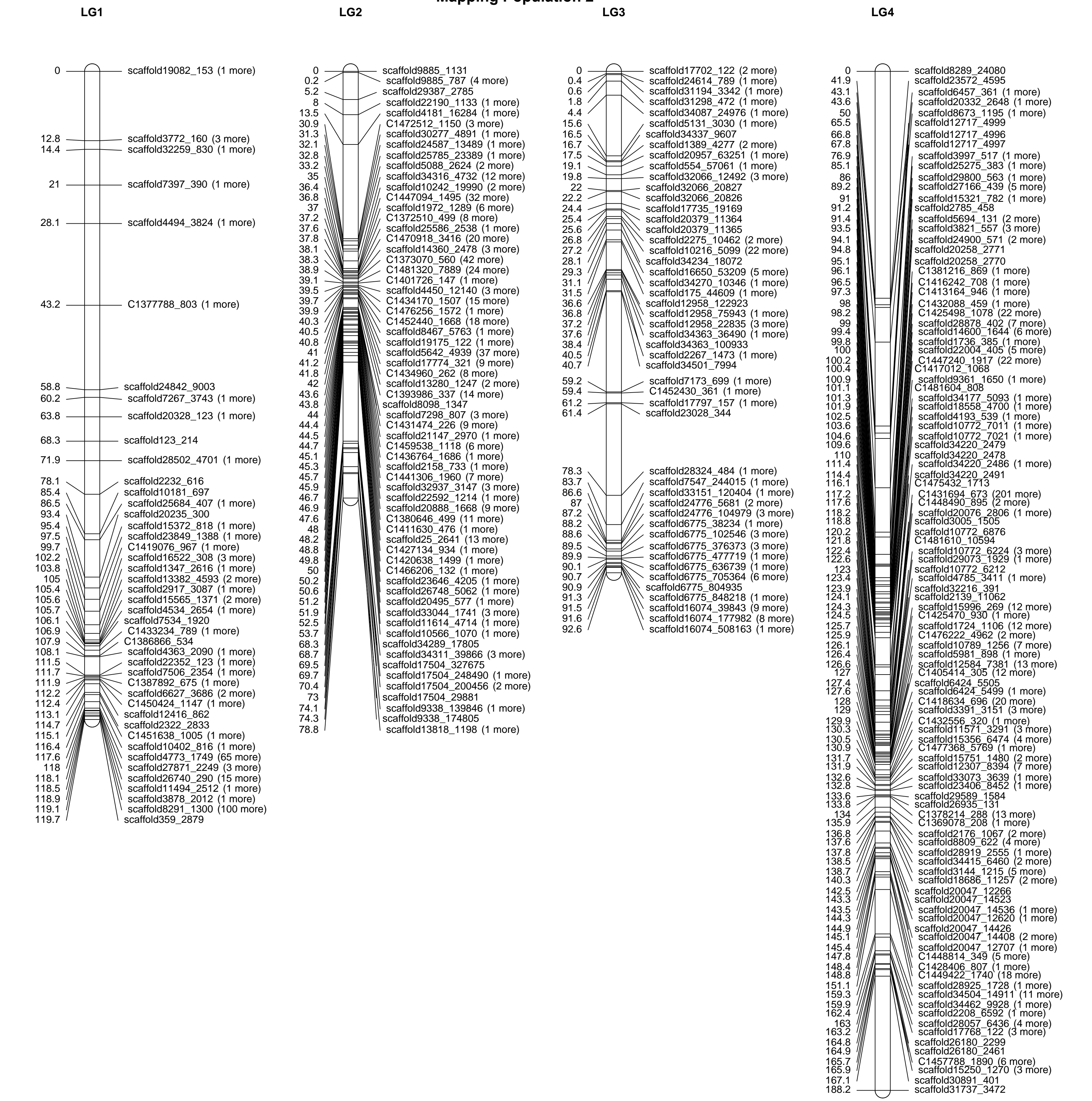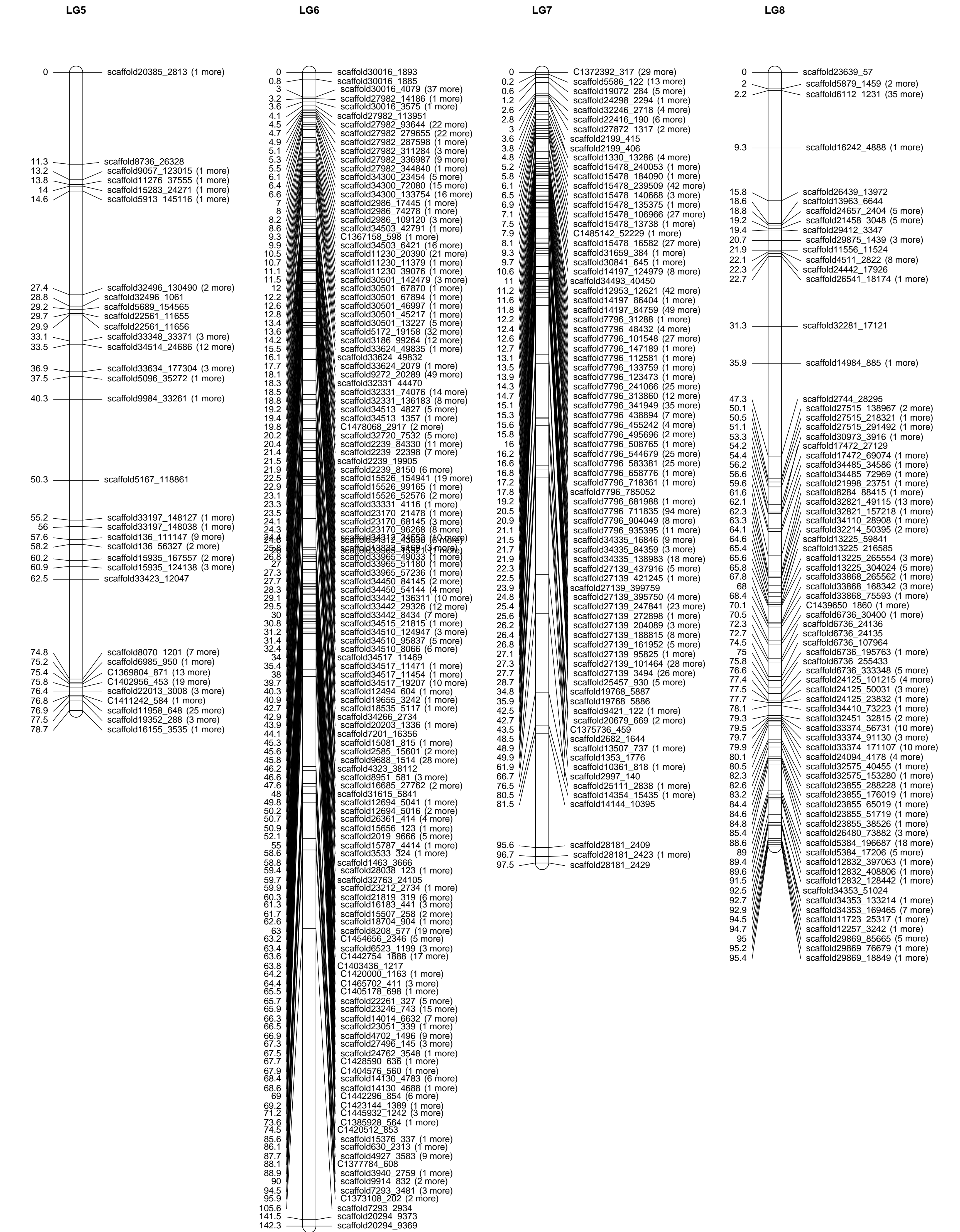

Supplement: Supplementary file 2 — Supplementary Material 2: Supplementary Fig. 1. PCoA plot based on SNP allele displaying genetic differences and similarities among the progenies and their respective founding parents of three field cress mapping populations MPs): MP1 MP2 and MP3. [file 12870_2025_6197_MOESM2_ESM.pdf]

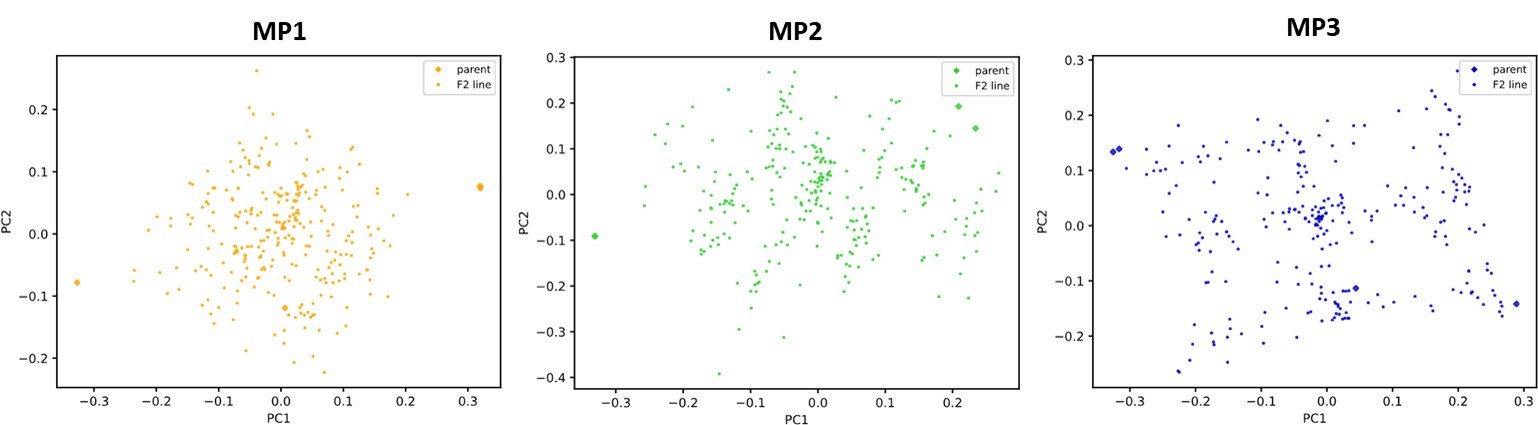

Supplement: Supplementary file 3 — Supplementary Material 3: Supplementary Fig.2. Genetic linkage of three bi-parental mapping populations of field cress. [file 12870_2025_6197_MOESM3_ESM.jpeg]

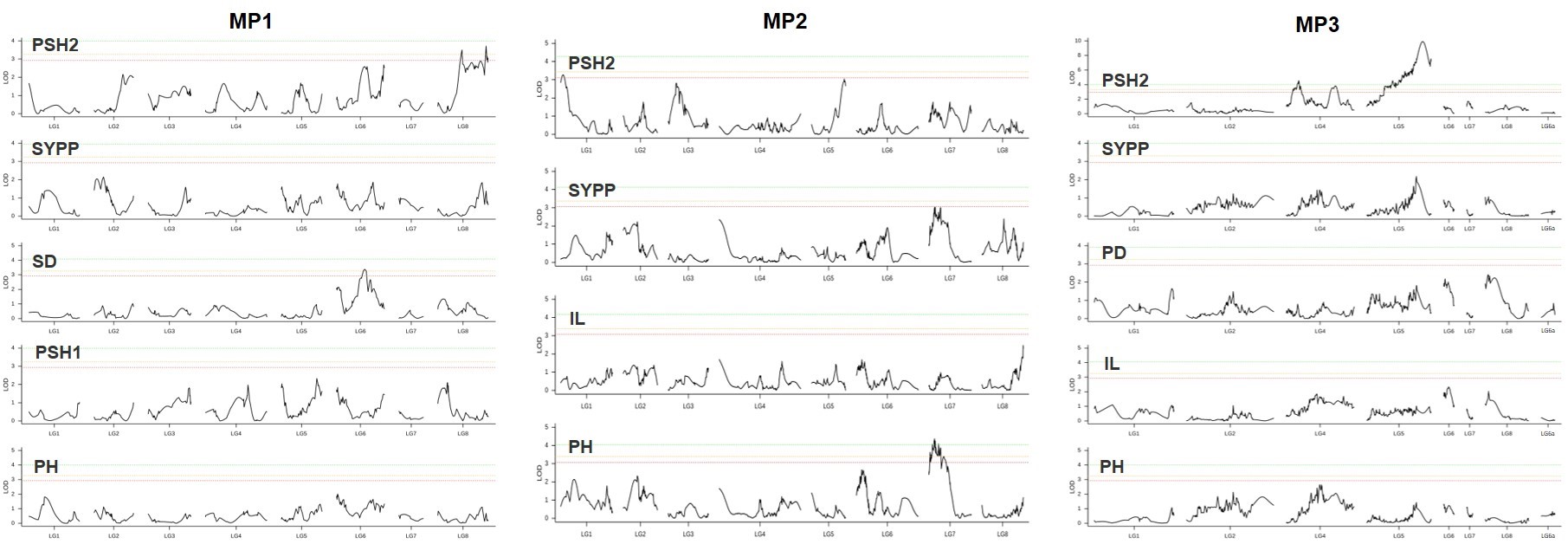

Supplement: Supplementary file 4 — Supplementary Material 4: Supplementary Fig.3. Multiple QTL interval mapping in three bi-parental mapping populations of field cress. (A) Mapping population 1, MP1; (B) Mapping population 2, MP2; (C) Mapping population 3, MP3. Plant height (PH), pod shattering (PSH1 and PSH2), seed yield per plant (SYPP), inflorescence length (IL), and seed dormancy (SD). The dotted lines indicate LOD score thresholds (red: P = 0.1, orange: P = 0.05, green: P = 0.01). [file 12870_2025_6197_MOESM4_ESM.jpeg]

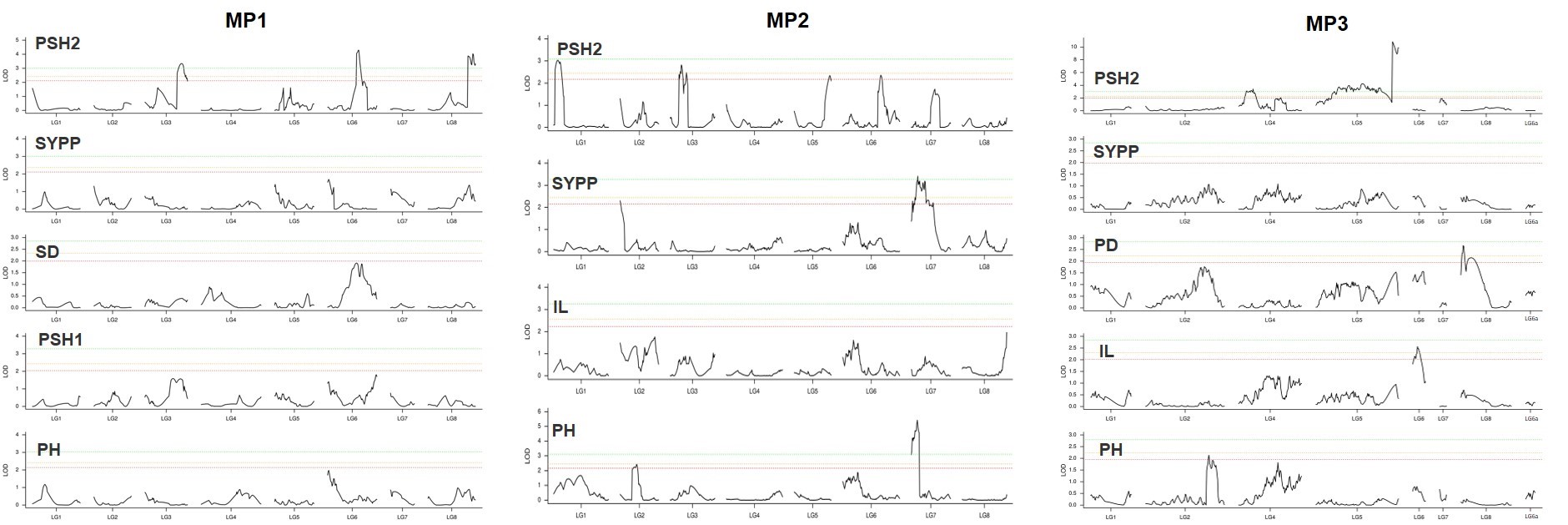

Supplement: Supplementary file 5 — Supplementary Material 5: Supplementary Fig.4. QTL interval mapping in three bi-parental mapping populations of field cress. (A) Mapping population 1, MP1; (B) Mapping population 2, MP2; (C) Mapping population 3, MP3. Plant height (PH), pod shattering (PSH1 and PSH2), seed yield per plant (SYPP), inflorescence length (IL), and seed dormancy (SD). The dotted lines indicate LOD score thresholds (red: P = 0.1, orange: P = 0.05, green: P = 0.01). [file 12870_2025_6197_MOESM5_ESM.jpeg]
